# The subtribes and genera of the tribe Listroderini (Coleoptera, Curculionidae, Cyclominae): Phylogenetic analysis with systematic and biogeographical accounts

**DOI:** 10.3897/zookeys.273.4116

**Published:** 2013-02-28

**Authors:** Juan J. Morrone

**Affiliations:** 1 Museo de Zoología “Alfonso L. Herrera”, Departamento de Biología Evolutiva, Facultad de Ciencias, Universidad Nacional Autónoma de México (UNAM), Apartado Postal 70-399, 04510 Mexico D.F., Mexico

**Keywords:** Cyclominae, weevils, Americas, Australia, New Zealand, Tristan da Cunha-Gough islands

## Abstract

The phylogenetic relationships of the genera of Listroderini LeConte, 1876 are analyzed based on 58 morphological characters. The genera are grouped in four clades, which are given subtribal status: Macrostyphlina new subtribe (*Adioristidius*, *Amathynetoides*, *Andesianellus*, *Macrostyphlus*, *Nacodius* and *Puranius)*, Palaechthina Brinck, 1948 (*Anorthorhinus*, *Gunodes*, *Haversiella*, *Inaccodes*, *Listronotus*, *Neopachytychius*, *Palaechthus*, *Palaechtodes*, *Steriphus* and *Tristanodes*), Falklandiina new subtribe (*Falklandiellus*, *Falklandiopsis*, *Falklandius*, *Gromilus*, *Lanteriella*, *Liparogetus*, *Nestrius* and *Telurus*), and Listroderina (*Acroriellus*, *Acrorius*, *Acrostomus*, *Antarctobius*, *Germainiellus*, *Hyperoides*, *Lamiarhinus*, *Listroderes*, *Methypora*, *Philippius*, *Rupanius* and *Trachodema*). The subtribes are characterized and keys to identify them and their genera are provided. Listroderini have four main biogeographical patterns: Andean (Macrostyphlina), Andean-New Zealand (Falklandiina), Andean-Neotropical-Australian (Listroderina) and Andean-Neotropical-Australian-New Zealand-Nearctic-Tristan da Cunha-Gough islands (Palaechthina). Geographical paralogy, particularly evident in the Subantarctic subregion of the Andean region, suggests that Listroderini are an ancient Gondwanic group, in which several extinction events might have obscured relationships among the areas.

## Introduction

Listroderini LeConte, 1876 are one of the largest tribes of Cyclominae (Oberprieler 2010, in press). They are widely distributed in the Southern Hemisphere, with the genus *Listronotus* also occurrying in North America (Morrone 2011) and fossils known from Antarctica (Ashworth and Kuschel 2003). The tribe was originally proposed by LeConte (1876) for the New World genera *Listroderes*, *Listronotus* and *Macrops*. In the following decades additional new taxa were described from Chile (Germain 1895–1896; Kuschel 1949, 1950, 1952), Argentina (Enderlein 1907, 1912; Hustache 1926), North and Central America (e.g., Henderson 1940; O’Brien 1977, 1981) and Peru (Voss 1954). Kuschel (1950, 1952, 1955) transferred some listroderine species to genera of Entiminae. Additionally, the circumscription of the tribe was expanded, because several genera that have been originally assigned to other tribes (and even subfamilies) from Australia (Erichson 1842; Pascoe1865, 1870; Blackburn 1890; Lea 1928), New Zealand (Broun 1893a, b, 1909, 1913, 1915) and the Tristan da Cunha-Gough islands (Brinck 1948) were transferred to Listroderini (Kuschel 1962, 1964, 1971, 1986; May 1994; Zimmerman 1994; Morrone 1997a). Recently, Oberprieler (2010) transferred *Rhigopsidius* from Rhythirrinini to Listroderini and reassigned the listroderine genus *Telurus* to the tribe Cylydrorhinini (Entiminae). According to the last checklist (Morrone 2011), a total of 407 species classified into 36 genera are assigned to Listroderini. Due to all these changes the taxa currently assigned to Listroderini constitute an assemblage that is difficult to characterize, and there is no complete treatment of all the genera.

Listroderini were originally assigned to the subfamily Cylydrorhininae (e.g., Enderlein 1907, 1912; Hustache 1926; Schenkling and Marshall 1931; Voss 1954; Kuschel 1955, 1958; O’Brien and Wibmer 1982). Kuschel (1964) transferred Listroderini to Rhyparosominae, which Kuschel (1971) later treated as a synonym of Rhythirrininae, and was followed by several authors (e.g., Wibmer and O’Brien 1986; Morrone et al. 1992; Morrone 1997a). Later, Rhythirrininae were demoted to a tribe of Cyclominae (Morrone 1997b), and thus Listroderini were considered as a subtribe (Anderson and Morrone 1996; Morrone 1997a, 2002a; Anderson 2002). More recently, Oberprieler (2010), while analysing the circumscription of Cyclominae and their tribes, reassigned tribal status to the listroderines.

Morrone (1997a) undertook a cladistic analysis of the South American genera of the tribe, considering that they represented a paraphyletic group, because the genera from Australia, New Zealand and the Tristan da Cunha-Gough islands are probably closely related to some of the American genera. The phylogenetic placement of these genera is not known, and the inclusion of *Rhigopsidius* and the exclusion of *Telurus* from the tribe, proposed by Oberprieler (2010), need to be tested.

My objective is to analyse the cladistic relationships of the genera of Listroderini, especially to determine the phylogenetic placement of the genera from Australia, New Zealand and the Tristan da Cunha-Gough islands. I intend to provide a phylogenetic framework for future studies and to summarize the systematics and biogeography of the genera to date.

## Material and methods

The studied specimens were provided by the following collections:

AMNH American Museum of Natural History, New York, USA.

AMPC Amyan MacFadyen, private collection, Coleraine, Northern Ireland.

ARPC Alexander Riedel, private collection, Friedberg, Germany.

BMNH The Natural History Museum, London, England.

BPBM Bernice P. Bishop Museum, Honolulu, USA.

CADIC Centro Austral de Investigaciones Científicas, Ushuaia, Argentina.

CBPC Carlos Bordón, private collection, Maracay, Venezuela.

CMNC Canadian Museum of Nature, Ottawa, Canada.

CNCI Canadian National Collection of Insects, Arachnids and Nematodes, Agriculture and Agri-Food Canada, Ottawa, Canada.

CWOB Charles W. O’Brien private collection, Arizona, USA.

DEI Deutsches Entomologisches Institut, EberswaldeFinow, Germany.

DZUP Departamento de Zoologia, Universidade Federal do Paraná, Curitiba, Brazil.

FIML Fundación e Instituto Miguel Lillo, San Miguel de Tucumán, Argentina.

FMNH Field Museum of Natural History, Illinois, USA.

GJWC Guillermo J. Wibmer, private collection, Tallahassee, USA.

IADIZA Instituto Argentino de Investigaciones de las Zonas Áridas, Mendoza, Argentina.

ICNB Instituto de Ciencias Naturales, Universidad Nacional de Colombia, Santafé de Bogotá, Colombia.

IPUM Instituto de la Patagonia, Universidad de Magallanes, Punta Arenas, Chile.

MACN Museo Argentino de Ciencias Naturales “Bernardino Rivadavia”, Buenos Aires, Argentina.

MCZ Museum of Comparative Zoology, Harvard University, Massachusetts, USA.

MHNS Museo Nacional de Historia Natural, Santiago, Chile.

MLP Museo de La Plata, La Plata, Argentina.

MNHN Museum National d´Histoire Naturelle, Paris, France.

MZFC Museo de Zoología “Alfonso L. Herrera”, Facultad de Ciencias, UNAM, Mexico City, Mexico.

NZAC New Zealand Arthropod Collection, Auckland, New Zealand.

SMTD Staatliches Museum für Tierkunde, Dresden, Germany.

USNM National Museum of Natural History, Washington D.C., USA.

ZMC Zoologisk Museum, Copenhagen, Denmark.

ZMHU Zoologische Museum der Humboldt Universität, Berlin, Germany.

Habitus drawing were made with a camera lucida attached to a stereoscopic microscope. Photographs were taken using a Scanning Electron Microscope at the Facultad de Ciencias, UNAM.

For the present study I examined species of the genera previously recognized for the tribe (Morrone 2011). The outgroup taxa included the genera *Hyomora* (Hipporhinini), *Aphela* (Notiomimetini), *Rhythirrinus* (Rhythirrinini) and *Telurus* (Cylydrorhinini). *Epicthonius* (Cyclomini) was used to root the cladograms.

The 58 morphological characters used in the analysis were taken from external structures (53) and male and female genitalia (5). The distribution of character states is shown in the data matrix (Table 1). The characters and their corresponding character states are as follows:

1 Body: length. (0) large to very large (> 15.0 mm); (1) medium-sized (7.1–14.9 mm); (2) small to very small (< 7.0 mm) [additive].

2 Vestiture: scales. (0) present; (1) absent.

**Figures 1–12.**
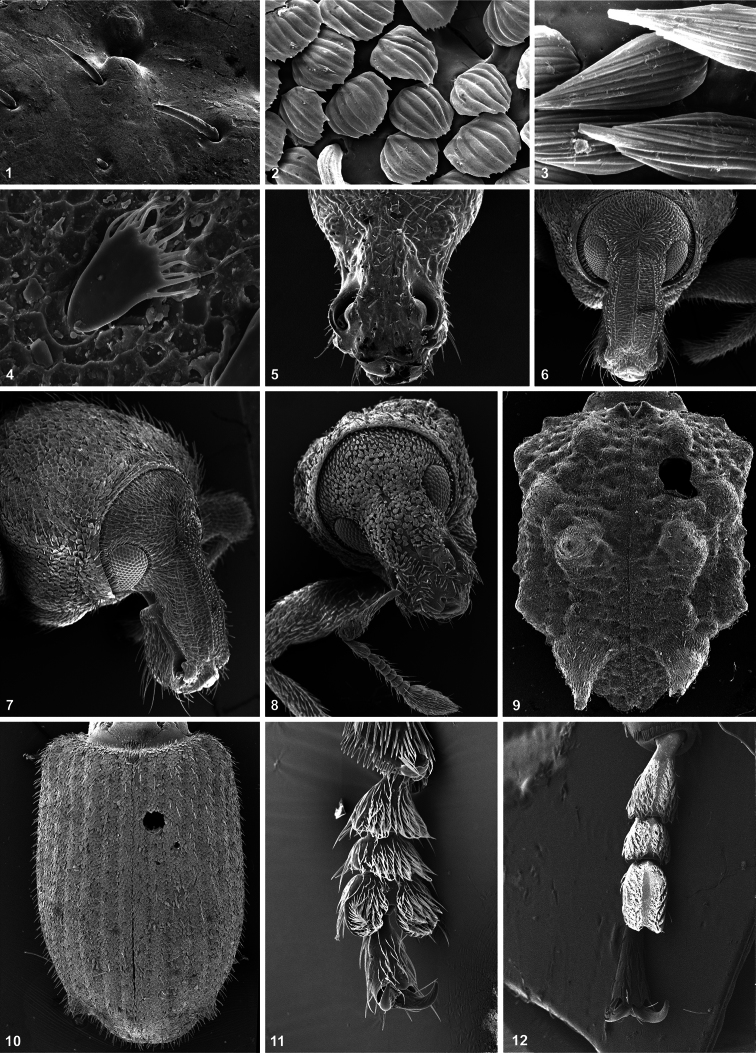
Some of the characters analysed. **1** Seta-like scales **2** subcircular scales **3** lanceolate scales **4** scales with finger-like processes **5, 6, 8** face and rostrum, dorsal view **7** face and rostrum, lateral view **8, 9** elytra, dorsal view **11, 12** tarsomere 3, ventral view. **1, 5** *Falklandius antarcticus*; **2, 8, 11** *Falklandiellus suffodens*; **3** *Hyperoides subcinctus*; **4, 12** *Philippius superbus*; **6, 7, 10** *Listroderes costirostris*; **9** *Lamiarhinus aelficus*.

3 Vestiture: scale shape. (0) seta-like (Fig. 1); (1) subcircular (Fig. 2); (2) lanceolate (Fig. 3); (3) with finger-like processes (Fig. 4) [non-additive].

4 Vestiture: setae. (0) present; (1) absent.

5 Rostrum: shape. (0) stout, very short (Fig. 5); (1) relatively stout, medium-sized, shorter than pronotum (Fig. 6); (2) slender, as long as or longer than pronotum [additive].

6 Rostrum: dorsal carinae. (0) present (Fig. 6); (1) absent (Figs 5, 8).

7 Scrobes: shape. (0) long, deep, sharply bordered, reaching eyes; (1) short, ill-defined, broad.

8 Epistome. (0) poorly demarcated; (1) raised.

9 Scrobes: position. (0) dorsolateral to dorsal; (1) lateral.

10 Suprascrobal keels. (0) absent; (1) present.

11 Scrobes: ventral tooth. (0) absent; (1) present (Fig. 7).

12 Pterygia. (0) simple, not exposed (Fig. 6); (1) auriculate, exposed (Fig. 5).

13 Mandibles. (0) with one apical cusp; (1) with two apical cusps.

14 Mandible and pharyngeal processes. (0); short and strong; (1) long and narrow.

15 Mandibles. (0) plurisetose (more than 4 setae); (1) paucisetose (1-4 setae).

16 Maxillary malae: teeth. (0) present; (1) absent.

17 Eyes: shape. (0) subcircular (Fig. 5); (1) transverse (Fig. 7).

18 Eyes: size. (0) large to medium (more than 30 facets); (1) small (10-25 facets); (2) very small (8 or fewer facets) [additive].

19 Eyes: position. (0) lateral (Fig. 6); (1) dorsal (Fig. 5).

20 Eyes: convexity. (0) strong; (1) slight; (2) flat [additive].

21 Antennal insertions. (0) distal; (1) at the middle of the rostrum.

22 Scapes: length. (0) long (surpassing posterior margin of eyes when resting in scrobe); (1) medium-sized (reaching eyes when resting in scrobe); (2) short (not reaching anterior margin of eyes when resting in scrobe) [additive].

23 Funicles: segment 1. (0) elongate; (1) globose.

24 Funicles: segments 2. (0) elongate; (1) globose.

25 Funicles: relative lengths of segments 1 and 2. (0) 1 longer than 2 (Fig. 8); (1) 1 subequal to or shorter than 2.

26 Funicles: segments 3–6. (0) elongate; (1) globose (Fig. 8).

27 Clubs: shape. (0) fusiform; (1) inflated.

28 Pronotum: shape. (0) subcircular; (1) transverse; (2) subtrapezoidal; (3) subquadrate; (4) subclyndrical [non-additive].

29 Pronotum: width. (0) larger than that of elytra; (1) smaller than that of elytra.

30 Pronotum: disc. (0) rugose; (1) smooth, polished.

31 Pronotum: tubercles. (0) absent; (1) present.

32 Postocular lobes. (0) present, well-developed; (1) present, slightly developed; (2) absent [additive].

33 Prosternum. (0) non-excavate; (1) excavate.

34 Metanepisternal sutures. (0) posteriorly fused or obliterated; (1) present, complete.

35 Scutellum. (0) not visible; (1) visible.

36 Elytra: shape. (0) oblong-oval (Fig. 10); (1) subrectangular (Fig. 9); (2) elongate-oval [non-additive].

37 Elytra. (0) not fused; (1) fused along interelytral suture.

38 Elytral disc. (0) convex; (1) slightly convex; (2) flat [additive].

39 Elytral intervals. (0) convex; (1) flat.

40 Elytral basal margin. (0) not raised; (1) raised, subcarinate.

41 Elytral humeri. (0) rounded; (1) subquadrate.

42 Elytral humeral tubercles. (0) absent; (1) present.

43 Several tubercles on elytral disc. (0) present, small, rounded; (1) absent; (2) present, strong (Fig. 9) [non-additive].

44 Series of three tubercles restricted to elytral interval 3. (0) absent; (1) present.

45 Series of declivital tubercles on elytra. (0) absent; (1) present.

46 Carina on elytral apical declivity. (0) absent; (1) present.

47 Anteapical elytral tubercle. (0) absent; (1) present.

48 Elytral apex, female. (0) not produced; (1) produced.

49 Femora: shape. (0) subcylindrical, clavate; (1) dorsoventrally compressed, clavate; (2) subcylindrical, markedly clavate [non-additive].

50 Tibiae: shape. (0) subcylindrical, laterally not expanded; (1) apically expanded.

51 Tibial spurs. (0) present; (1) absent.

52 Tarsomeres 3. (0) bilobed (Fig. 11); (1) subcylindrical (Fig. 12).

53 Ventrites 3 and 4, female. (0) combined shorter than 5; (1) combined longer than 5.

54 Aedeagus, lateral view. (0) robust; (1) slender.

55 Distal gonocoxites. (0) strongly sclerotized; (1) membranous.

56 Styli. (0) well-developed, claw-like; (1) well-developed, finger-like; (2) reduced to a few vibrissae [non-additive].

57 Apodeme of female sternum 8. (0) short (< 3 times longer than plate); (1) long (> 4 times longer than plate).

58 Plate of female sternum 8. (0) developed; (1) reduced.

The cladograms were constructed using software TNT (Goloboff et al. 2008). A first analysis was conducted treating all characters under equal weights. Then, the effect of homoplasy on the results was explored by conducting different implied weights analyses (Goloboff 1993), with constants of concavity (k) set to a different integer value of 1–12, where 1 is weighted most severely against homoplastic characters. Implied weights analyses were conducted using the heuristic “traditional search” algorithm of TNT, with 1000 replications and tree-bisection-reconnection branch-swapping (TBR), holding 1000 trees during each replication.

**Table 1.** Data matrix analysed. Character states of polymorphic taxa are indicated between square brackets.<br/>

*Epicthonius*	0000000000000000000000000000000000000000000000000000000000
*Hyomora*	1010000010001010100202000100000001000000001000000110000000
*Rhythirrinus*	1010000010001010100101000101001011110000001000100010000200
*Aphela*	21?0010010001010010201101100000001000000001000000010001200
*Telurus*	21?0011010011010000101000110000200100000001000010000101210
*Acroriellus*	2000101010001010100101000101000101100010000110000000001200
*Acrorius*	2000101010001010100101000101001101100100000010100000001100
*Acrostomus*	1000101110101010100100000003000101100010001000000000001[01]00
*Adioristidius*	2000100011001010110101000104000100100000001000000000011110
*Amathynetoides*	2000100011001010110101000100010101100010001000000000011110
*Andesianellus*	21?0100011001010020201000114000200100001001000000000001110
*Anorthorhinus*	2000200010001010100102000004000101100000001000000000001100
*Antarctobius*	10[01]0101010001010100101000100000201100000001000100000001[12]00
*Falklandiellus*	2010011010011010100101000101000200100000001010000000001100
*Falklandiopsis*	21?0011010011010101201000100000101100000011010002000001100
*Falklandius*	20?0011000011010011200010110000200100000001000000000001110
*Germainiellus*	1000101010001010100101000101000101100000001000100000001100
*Gromilus*	2000101010011010100200000004000101100000000000000000001100
*Gunodes*	1010200010001010100102001110000101100010001000000000001100
*Haversiella*	2011210010001011100112000100000201120010001000000010001201
*Hyperoides*	1020101010001010100100000101000101100010001000000000011100
*Inaccodes*	2000200010001010100102000110000101100010001000000000001100
*Lamiarhinus*	2000101010001010100100000001001101111000102010100000001100
*Lanteriella*	21?00110000110100212000101000102001000100010000011000?12?0
*Liparogetus*	1000001010011010100111000113000201100000001000000010001100
*Listroderes*	[12]010101010[01]01010100101000101000101100000001000100000001[01]00
*Listronotus*	[12]010[12]00010001010100101001104000101100000001000100000001100
*Macrostyphlus*	2010100011001010100101000114000200100000000000000000011110
*Methypora*	2010100010001010100102000104000101110200000000110000001100
*Nacodius*	2000100011001010100100000100010201100010001000000000011100
*Neopachytychius*	2010200010001110100102000100000101100010001000000000001100
*Nestrius*	2000101000011010010200000114000200000000001000000000001100
*Palaechthus*	1000210010001010100102001112000101120010001000000000001100
*Palaechtodes*	1000200010001010100102001114000101120010001000000000001100
*Philippius*	0030101010001010110201000100101101011200101010100011001100
*Puranius*	2010100011001010100101000111000101100010000000000000011110
*Rhigopsidius*	1030000110001010100201000101001011110000102010100000000000
*Rupanius*	2000100010001010100101000101001101110100002001000000001200
*Steriphus*	1010200010001010100100000100000101120000001000100000001100
*Trachodema*	2030101010001010100100000101001101100100102010100000001100
*Tristanodes*	2000200010001010100102001114000101120010001000000000001100

## Results

### Phylogenetic Analysis

**Figures 13–16.**
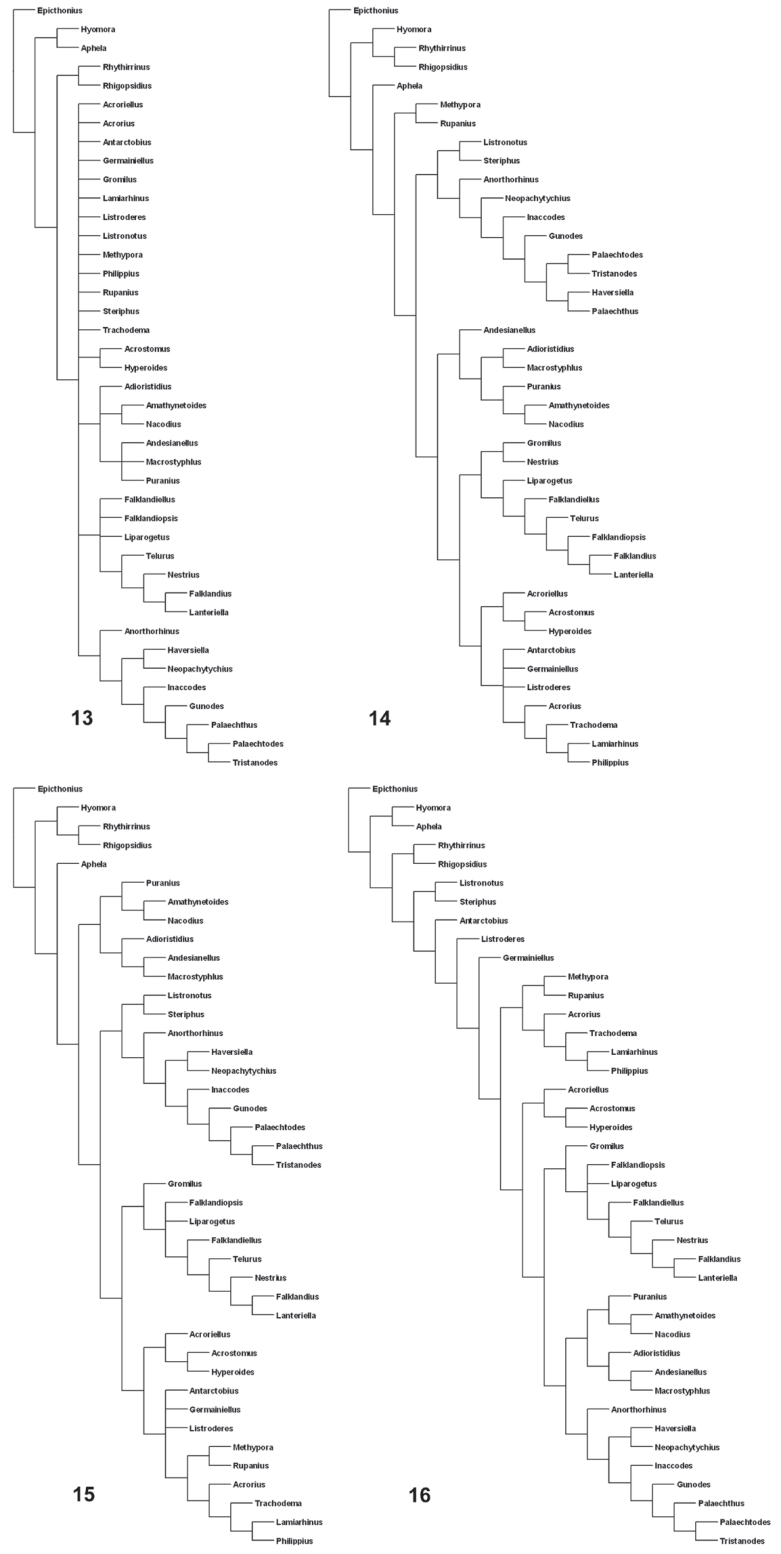
Consensus cladograms of the different analyses. **13** equal weights **14** k=3 **15** k=6 **16** k=12.

The analysis of the data matrix (Table 1) under equal weights and with different concavity constants led to different cladograms: 100 cladograms under equal weights (Fig. 13); three cladograms with k=3 (Fig. 14); six cladograms with k= 6 (Fig. 15); and two cladograms with k= 12 (Fig. 16). In all the analyses the tribe Listroderini is recovered as a monophyletic taxon. *Rhigopsidius*, previously placed by Oberprieler (2010) in Listroderini, resulted to be the sister taxon to *Rhythirrinus* (Rhythirrinini). *Telurus*, excluded from Listroderini by Oberprieler (2010), was placed within Listroderini. In the analyses with k=3 and 6, *Aphela* (Notiomimetini) is the sister taxon to Listroderini. In spite of the different results, there are some larger clades that were fairly constant.

**Figure 17.**
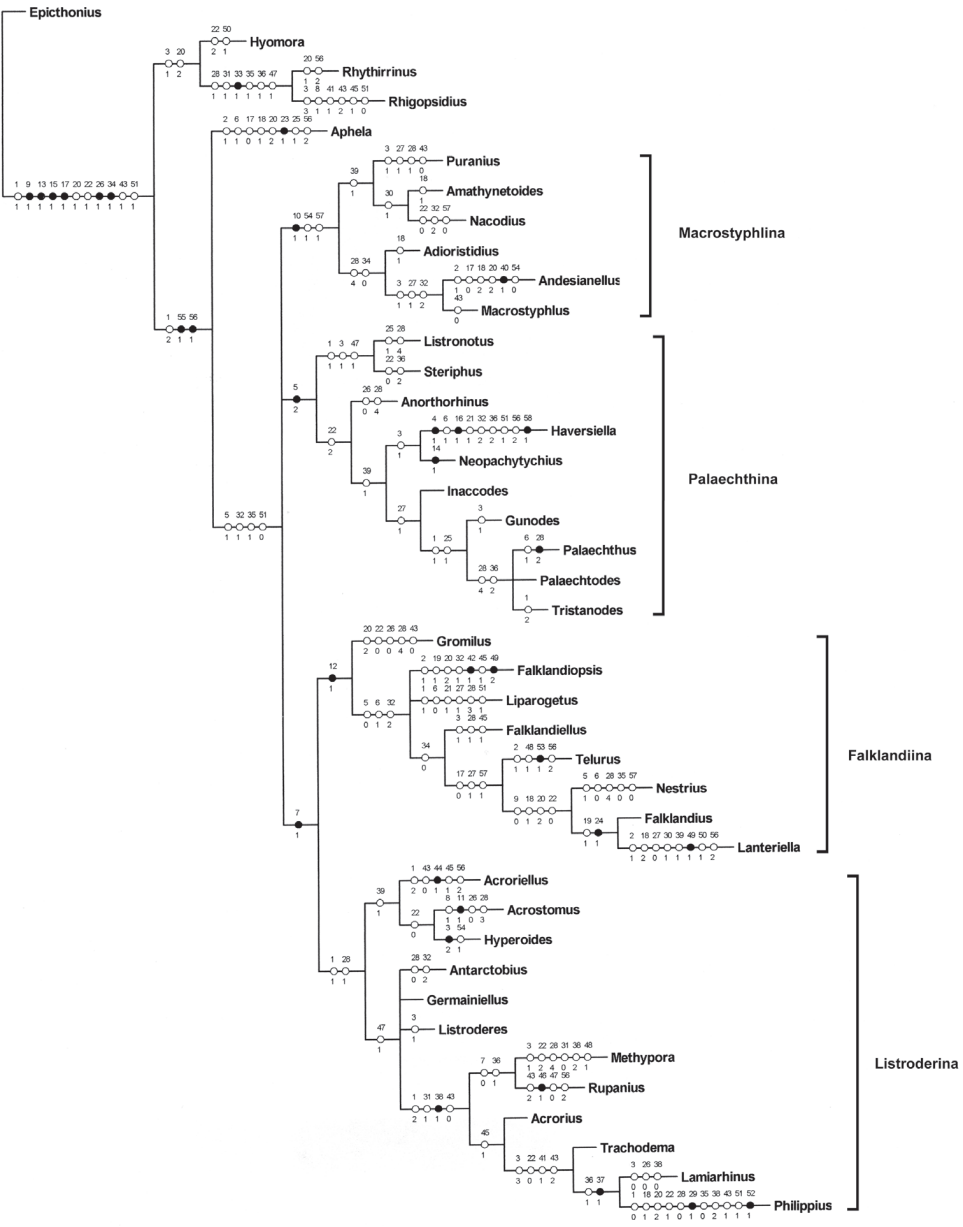
Consensus cladgrma of the cladograms obtained with k=6 with character state changes indicated.

I consider that the results of the analysis with k= 6 are not as extreme as the others and show more clearly the four main clades, which are treated herein as subtribes (Fig. 17):

1 Macrostyphlina new subtribe: genera *Adioristidius*, *Amathynetoides*, *Andesianellus*, *Macrostyphlus*, *Nacodius* and *Puranius*.

2 Palaechthina Brinck, 1948: genera *Anorthorhinus*, *Gunodes*, *Haversiella*, *Inaccodes*, *Listronotus*, *Neopachytychius*, *Palaechthus*, *Palaechtodes*, *Steriphus* and *Tristanodes*.

3 Falklandiina new subtribe: genera *Falklandiellus*, *Falklandiopsis*, *Falklandius*, *Gromilus*, *Lanteriella*, *Liparogetus*, *Nestrius* and *Telurus*.

4 Listroderina LeConte, 1876: genera *Acroriellus*, *Acrorius*, *Acrostomus*, *Antarctobius*, *Germainiellus*, *Hyperoides*, *Lamiarhinus*, *Listroderes*, *Methypora*, *Philippius*, *Rupanius* and *Trachodema*.

### Systematic Account

#### Tribe Listroderini

LeConte, 1876

Figs 18 -35

Listroderi LeConte, 1876: 124.
Listroderitos Germain, 1895: 287.
Listroderina Champion, 1902: 120.
Listroderini Hustache, 1926: 175.
Listroderinae Thompson, 1992: 876.

##### Type genus.

*Listroderes* Schönherr, 1826.

##### Diagnosis. 

Very small to very large (1.0–22.8 mm); integument reddish brown (black in *Acrostomus*); vestiture consisting mostly of dense scales and setae (rarely only scales or setae), setae on rostrum and pronotum directed anteriad or mesad, on elytra posteriad; rostrum stout and very short to slender, as long as or longer than pronotum; scrobes usually lateral; epistome poorly demarcated, rarely raised (*Acrostomus*); eyes usually large, flat, transverse or subcircular; mandibles with two apical cusps and paucisetose (1-4 setae); antennae with funicle 7-segmented, segments 1 and usually 2 elongate, clubs fusiform or inflated; prothorax with or without postocular lobes; prosternum long, non-excavate; elytra oblong-oval, elongate-oval or subrectangular; tibiae mucronate, generally with spurs (when present pro- and mesotibiae with 1 spur and metatibiae with 1–2 spurs); claws divaricate, simple or with slight basal swelling; aedeagus with tegmen lacking parameres (reduced in *Methypora*); distal gonocoxites membranous, generally simple, with large, apical or subapical stylus carrying a tuft of setae, but occasionally without stylus and apex of gonocoxite flattened and bent outwards.

##### Comparative notes.

Listroderini were formerly considered as related to Rhythirrinini (Kuschel 1971; Anderson and Morrone 1996; Morrone 1997a, b, 2002a; Anderson 2002). Oberprieler (2010, in press) considered Notiomimetini to be close relatives of Listroderini, although he suggested that more detailed studies would be required to decide whether they should be merged into a single tribe or not. Based on the results of this analysis, Listroderini and Notiomimetini *(Aphela)* are hypothesized to be sister tribes.

##### Biology.

Larvae of Listroderini are generally oligophagous ectophytic root-feeders (Oberprieler in press). Adults feed on the leaves of a variety of angiosperms (Morrone 2011).

##### Key to the subtribes of Listroderini

**Table:** 

1	Rostrum slender, as long as or longer than pronotum (except shorter than pronotum in some species of *Listronotus*); scrobes long, sharply bordered, reaching eyes; funicular segment 1 usually subequal to or shorter than 2; commonly associated with aquatic or semiaquatic plants	Palaechthina
–	Rostrum stout or relatively stout, shorter than pronotum; scrobes usually short, ill-defined, broad; funicular segment 1 longer than 2; associated to terrestrial plants	2
2	Rostral dorsal carinae usually absent; pterygia auriculate, exposed (Fig. 5)	Falklandiina
–	Rostral dorsal carinae present; pterygia simple, not exposed (Fig. 6)	3
3	Scrobes short, ill-defined, broad, lacking suprascrobal keel; elytra with intervals convex, with anteapical tubercle (except for *Rupanius*)	Listroderina
–	Scrobes long, deep, sharply bordered, reaching eyes, with suprascrobal keel; elytra with intervals usually flat, lacking anteapical tubercle	Macrostyphlina

#### Macrostyphlina subtr. n.

##### Type genus.

*Macrostyphlus* Kirsch, 1889.

##### Diagnosis.

Scrobes long, deep, sharply bordered, reaching eyes, with suprascrobal keel; elytra oblong-oval, with intervals usually flat, lacking anteapical tubercle.

##### Included taxa.

This new subtribe, which basically corresponds to the *Macrostyphlus* generic group of Morrone (1994c, 1997a), includes the genera *Adioristidius*, *Amathynetoides*, *Andesianellus*, *Macrostyphlus*, *Nacodius* and *Puranius*. All these genera are distributed in South America, in the Andean region and the South American Transition Zone (*sensu* Morrone 2006).

##### Key to the genera of Macrostyphlina

**Table:** 

1	Postocular lobes present	2
–	Postocular lobes absent	4
2	Pronotum transverse to strongly transverse	*Puranius*(Fig. 19)
–	Pronotum subcircular or subcylindrical	3
3	Pronotum subcircular with subparallel flanks, disc smooth, polished; metanepisternal sutures present, complete; elytra with intervals flat	*Amathynetoides*
–	Pronotum subcylindrical, disc rugose; metanepisternal sutures posteriorly fused or obliterated; elytra with intervals convex	*Adioristidius*(Fig. 18)
4	Vestiture consisting of subcircular scales and setae; elytra with small, rounded tubercles	*Macrostyphlus*
–	Vestiture consisting of seta-like scales and setae or only setae; elytra lacking tubercles	5
5	Vestiture consisting of seta-like scales and setae; eyes large, slightly convex; pronotum disc smooth, polished; basal elytral margin not raised	*Nacodius*
–	Vestiture consisting of setae only; eyes very small, microphthalmic (8 or fewer facets), flat; pronotum disc rugose; basal elytral margin raised, subcarinate	*Andesianellus*

#### Adioristidius

Morrone, 1994

http://species-id.net/wiki/Adioristidius

Fig. 18

Adioristidius Voss, 1954: 242 (not available, type species not designated).
Anchadioristus Voss, 1954: 242 (not available, type species not designated).
Adioristidus Edwards & Hopwood, 1966: 5 (lapsus).
Adioristidius Morrone, 1994c: 13.

##### Type species.

*Adioristus similaris* Voss, 1954.

##### Diagnosis.

Small to very small (1.5–4.1 mm); vestiture consisting of seta-like scales and setae; antennal clubs fusiform; pronotum subcylindrical, disc rugose; metanepisternal sutures posteriorly fused or obliterated; elytral intervals convex.

##### Relationships.

*Adioristidius* is the sister genus of *Macrostyphlus-Andesianellus*.

##### Species included.

*Adioristidius anchonoideus* (Hustache, 1938); *Adioristidius carinicollis* (Voss, 1954); *Adioristidius chilensis* Morrone, 1994; *Adioristidius costulatus* (Hustache, 1938); *Adioristidius crassirostris* (Hustache, 1938); *Adioristidius cuprisquameus* (Voss, 1954); *Adioristidius granulatus* (Hustache, 1938); *Adioristidius hirsutus* Morrone, 1994; *Adioristidius hydanius* Morrone, 1994; *Adioristidius jorgei* Morrone, 1994; *Adioristidius lidiae* Morrone, 1994; *Adioristidius manu* Morrone, 1994; *Adioristidius morio* (Voss, 1954); *Adioristidius nivalis* (Kuschel, 1949); *Adioristidius pampaensis* (Voss, 1954); *Adioristidius peruvianus* (Voss, 1954); *Adioristidius puncticollis* (Hustache, 1938); *Adioristidius scrobicollis* (Voss, 1954); *Adioristidius similaris* (Voss, 1954); *Adioristidius subimpressus* (Voss, 1954); *Adioristidius subtuberculatus* (Voss, 1954); *Adioristidius sulcicollis* (Hustache, 1938); *Adioristidius tuberculatus* (Voss, 1954); *Adioristidius variegatus* (Voss, 1954).

##### Host plants.

*Adioristidius chilensis*: *Mulinum* spp. (Apiaceae); *Adioristidius tuberculatus*: *Solanum tuberosum* L. (Solanaceae) (Morrone 1994c).

##### Geographical distribution.

South American Transition Zone (Puna biogeographical province) and Central Chilean and Subantarctic subregions (Andean region), from Peru to Central Chile (Morrone 1994c).

##### Material examined.

*Adioristidius anchonoideus* (CMNC, DEI, MLP, MZFC), *Adioristidius chilensis* (MHNS), *Adioristidius costulatus* (DEI), *Adioristidius crassirostris* (DEI), *Adioristidius granulatus* (DEI), *Adioristidius hirsutus* (MHNS, MLP, MZFC), *Adioristidius hydanius* (DEI), *Adioristidius jorgei* (MHNS, MLP, MZFC), *Adioristidius lidiae* (CMNC), *Adioristidius manu* (CMNC, FMNH), *Adioristidius morio* (CWOB, MLP, MZFC), *Adioristidius nivalis* (MHNS, NZAC), *Adioristidius puncticollis* (DEI, MZFC), *Adioristidius similaris* (DEI), *Adioristidius sulcicollis* (DEI), *Adioristidius tuberculatus* (CWOB, MZFC, USNM), *Adioristidius variegatus* (DEI).

**Figures 18–26.**
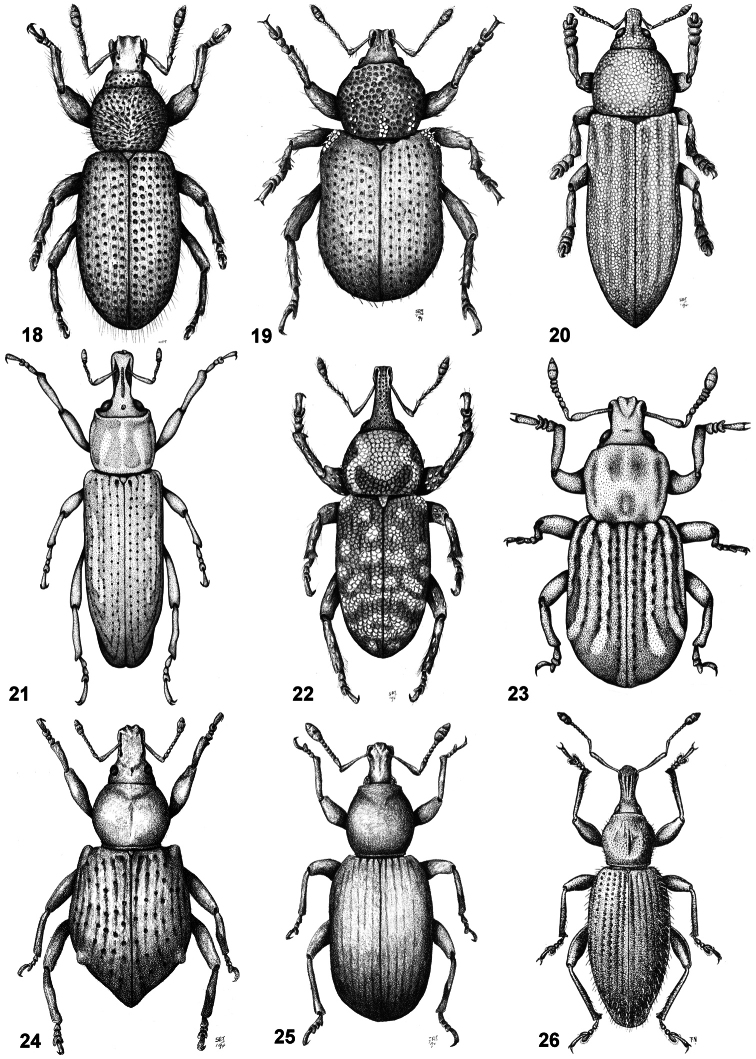
Habitus of representative Listroderini. **18** *Adioristidius hirsutus* **19** *Puranius nigrinus* **20** *Haversiella albolimbata* **21** *Listronotus bosqi* **22** *Neopachytychius squamosus* **23** *Falklandiellus suffodens* **24** *Falklandiopsis magellanica* **25** *Falklandius antarcticus* **26** *Gromilus veneris*.

#### Amathynetoides

Morrone, 1994

http://species-id.net/wiki/Amathynetoides

Amathynetes Kuschel, 1949: 43 (*non* Olliff, 1891; misidentification, in part).
Amathynetoides Morrone, 1994c: 28.

##### Type species.

*Amathynetes appendiculatus* Kuschel, 1949.

##### Diagnosis.

Small to very small (3.0–6.6 mm); vestiture consisting of seta-like scales and setae; pronotum subcircular with subparallel flanks, disc smooth, polished; metanepisternal sutures present, complete; elytral intervals flat.

##### Relationships.

*Amathynetoides* is the sister genus of *Nacodius*.

##### Species included.

*Amathynetoides appendiculatus* (Kuschel, 1949); *Amathynetoides ebeninus* (Hustache, 1938); *Amathynetoides intemperatus* Morrone, 1994; *Amathynetoides longulus* (Kuschel, 1949); *Amathynetoides morbeamus* Morrone, 1994; *Amathynetoides nitidiventris* (Hustache, 1938); *Amathynetoides normae* Morrone, 1994; *Amathynetoides palustris* (Kuschel, 1949); *Amathynetoides sparsesetosus* (Hustache, 1938); *Amathynetoides sundrianus* Morrone, 1994.

##### Host plants.

*Amathynetoides nitidiventris*: *Ullucus tuberosus* Caldas (Basellaceae) (López and Hermann 2004).

##### Geographical distribution.

South American Transition Zone (Puna and Coastal Peruvian Desert biogeographical provinces), from Peru to northern Chile (Morrone 1994c).

##### Material examined.

*Amathynetoides appendiculatus* (CWOB, CMNC, MHNS, MZFC, NZAC, USNM), *Amathynetoides ebeninus* (BPBM, CWOB, DEI, MZFC), *Amathynetoides intemperatus* (AMNH, CWOB, MLP, MZFC), *Amathynetoides longulus* (CWOB, MHNS, NZAC, MZFC, USNM), *Amathynetoides morbeamus* (FIML), *Amathynetoides nitidiventris* (DEI), *Amathynetoides normae* (CMNC, MLP, MZFC), *Amathynetoides palustris* (CWOB, FIML, MHNS, MZFC, NZAC, USNM), *Amathynetoides sparsesetosus* (CWOB, DEI, CMNC, MZFC), *Amathynetoides sundrianus* (BMNH, CWOB, FIML, MLP, MZFC).

#### Andesianellus

Anderson & Morrone, 1996

http://species-id.net/wiki/Andesianellus

Andesianellus Anderson & Morrone, 1996: 260.

##### Type species.

*Andesianellus microphthalmicus* Anderson & Morrone, 1996.

##### Diagnosis.

Very small (1.9–3.3 mm); vestiture consisting of setae only; eyes very small, (8 or fewer facets), flat; postocular lobes absent; basal elytral margin raised, subcarinate.

##### Relationships.

*Andesianellus* is the sister genus of *Macrostyphlus*, ashypothesizedin previous analyses (Anderson and Morrone 1996; Morrone 1997a).

##### Biology.

Species of this genus have been reported as leaf-litter inhabitants (Anderson and Morrone 1996).

##### Species included.

*Andesianellus carltoni* Anderson & Morrone, 1996; *Andesianellus cotopaxi* Anderson & Morrone, 1996; *Andesianellus fulgidus* Anderson & Morrone, 1996; *Andesianellus hermani* Anderson & Morrone, 1996; *Andesianellus masneri* Anderson & Morrone, 1996; *Andesianellus microphthalmicus* Anderson & Morrone, 1996; *Andesianellus minutus* Anderson & Morrone, 1996; *Andesianellus planirostris* Anderson & Morrone, 1996; *Andesianellus tricarinatus* Anderson & Morrone, 1996.

##### Geographical distribution.

South American Transition Zone (North Andean Paramo biogeographical province), in Colombia, Ecuador and Peru (Anderson and Morrone 1996).

##### Material examined.

*Andesianellus carltoni* (CMNC), *Andesianellus cotopaxi* (AMNH), *Andesianellus fulgidus* (CMNC), *Andesianellus hermani* (AMNH), *Andesianellus masneri* (CMNC), *Andesianellus microphthalmicus* (CMNC, MLP), *Andesianellus minutus* (CMNC, FMNH), *Andesianellus planirostris* (AMNH, BMNH, CMNC, CWOB, FMNH, MLP, USNM), *Andesianellus tricarinatus* (CMNC, FMNH).

#### Macrostyphlus

Kirsch, 1889

http://species-id.net/wiki/Macrostyphlus

Macrostyphlus Kirsch, 1889: 25.

##### Type species.

*Macrostyphlus gualcalae* Kirsch, 1889 (by indication, monotypy).

##### Diagnosis.

Very small (1.9–3.5 mm); vestiture consisting of subcircular scales and setae; pronotum subclyndrical; metanepisternal sutures posteriorly fused or obliterated; elytra with intervals convex.

##### Relationships.

*Macrostyphlus* is the sister genus of *Andesianellus*, ashypothesized in a previous analysis (Morrone 1997a).

##### Species included.

*Macrostyphlus bilbo* Morrone, 1994; *Macrostyphlus coelorum* (Olliff, 1891); *Macrostyphlus frodo* Morrone, 1994; *Macrostyphlus gandalf* Morrone, 1994; *Macrostyphlus gualcalae* Kirsch, 1889; *Macrostyphlus howdenorum* Morrone, 1994; *Macrostyphlus peruvianus* Morrone, 1994; *Macrostyphlus sturmi* Morrone, 1994; *Macrostyphlus transatlanticus* (Kirsch, 1889); *Macrostyphlus venezolanus* Morrone, 1994.

##### Geographical distribution.

South American Transition Zone (North Andean Paramo and Puna biogeographical provinces), from eastern Venezuela to southern Peru (Morrone 1994c).

##### Material examined.

*Macrostyphlus bilbo* (CNCI), *Macrostyphlus coelorum* (CWOB), *Macrostyphlus frodo* (ICNB, USNM), *Macrostyphlus gandalf* (CMNC, CNCI, MLP, MZFC), *Macrostyphlus gualcalae* (SMTD), *Macrostyphlus howdenorum* (CMNC), *Macrostyphlus peruvianus* (FMNH), *Macrostyphlus sturmi* (ICNB), *Macrostyphlus transatlanticus* (SMTD), *Macrostyphlus venezolanus* (MZFC).

#### Nacodius

Morrone, 1994

http://species-id.net/wiki/Nacodius

Nacodius Morrone, 1994e: 3.

##### Type species.

*Nacodius martitae* Morrone, 1994.

##### Diagnosis.

Small (4.6–6.9 mm); vestiture of seta-like scales and setae; eyes large, slightly convex; pronotum lacking postocular lobes, with disc smooth, polished; elytra with intervals flat.

##### Relationships.

*Nacodius* is the sister genus to *Amathynetoides*, and both are placed in Macrostyphlina. In a previous analysis (Morrone 1997a) *Nacodius* was placed in the *Antarctobius* generic group (= Listroderina).

##### Species included.

*Nacodius alectrus* Morrone, 1994; *Nacodius brevirostris* (Voss, 1954); *Nacodius martitae* Morrone, 1994; *Nacodius omissus* (Kuschel, 1952).

##### Geographical distribution.

South American Transition Zone (North Andean Paramo and Puna biogeographical provinces), in Ecuador and Peru (Morrone 1994e).

##### Material examined.

*Nacodius alectrus* (CWOB), *Nacodius brevirostris* (SMTD), *Nacodius martitae* (AMNH, CWOB, MLP, MZFC) and *Nacodius omissus* (BMNH).

#### Puranius

Germain, 1895

http://species-id.net/wiki/Puranius

Fig. 19

Puranius Germain, 1895: 313.
Puranus Germain, 1911: 205 (lapsus).
Reichertia Enderlein, 1912: 31 (type species: *Listroderes sculpticollis* Enderlein, 1907, by original designation).

##### Type species.

*Puranius inaequalis* Germain, 1896 (subsequent designation by Morrone, 1994c).

##### Relationships.

*Puranius* is the sister genus to *Amathynetoides-Nacodius*.

##### Diagnosis.

Small to very small (1.9–6.5 mm); vestiture of subcircular scales and setae; pronotum transverse to strongly transverse; metanepisternal suture present, complete; elytra oblong-oval, with small, rounded tubercles.

##### Species included.

*Puranius argentinensis* Morrone, 1994; *Puranius australis* Germain, 1896; *Puranius championi* (Kuschel, 1952); *Puranius dubius* (Germain, 1896); *Puranius elguetai* Morrone, 1994; *Puranius exsculpticollis* (Enderlein, 1907); *Puranius fasciculiger* (Blanchard, 1851); *Puranius hispidus* (Germain, 1896); *Puranius inaequalis* Germain, 1896; *Puranius midas* Morrone, 1994; *Puranius nigrinus* (Fairmaire, 1884); *Puranius obrienorum* Morrone, 1994; *Puranius pusillus* Morrone, 1994; *Puranius scaber* (Enderlein, 1907); *Puranius sylvanius* Morrone, 1994; *Puranius torosus* Morrone, 1994; *Puranius tothus* Morrone, 1994; *Puranius tuberosus* Germain, 1896; *Puranius verrucosus* (Germain, 1896); *Puranius vulgaris* Morrone, 1994.

##### Host plants.

*Puranius argentinensis*:*Mulinum* sp. (Apiaceae); *Puranius championi*: *Poa flabellata* (Lam.) Raspail (Poaceae); *Puranius fasciculiger*: *Senecio smithii* DC (Asteraceae); *Puranius nigrinus*: *Taraxacum officinale* Weber ex F. H. Wigg. (Asteraceae) and *Nothofagus* sp. (Nothofagaceae); *Puranius vulgaris*: *Mulinum* sp. (Apiaceae); *Puranius scaber*: *Baccharis* sp. (Asteraceae) and *Ephedra* sp. (Ephedraceae) (Morrone, 1994c).

##### Geographical distribution.

Andean region (Subantarctic and Central Chilean subregions) and South American Transition Zone, from southern Argentina, including the Falkland Islands (Islas Malvinas), to Peru (Morrone 1994c; Posadas 2008, 2012).

##### Material examined.

*Puranius argentinensis* (AMNH, BMNH, MLP, MZFC), *Puranius australis* (AMNH, CWOB, MHNS, NZAC), *Puranius championi* (BMNH, CWOB, NZAC), *Puranius dubius* (CWOB, MHNS, NZAC), *Puranius elguetai* (AMNH, MHNS, MLP, MZFC), *Puranius exsculpticollis* (BMNH), *Puranius fasciculiger* (CWOB, MHNS, NZAC, USNM), *Puranius hispidus* (CWOB, MHNS, NZAC), *Puranius inaequalis* (CMNC, CWOB, MHNS, MZFC, NZAC), *Puranius midas* (AMNH), *Puranius nigrinus* (ARPC, BMNH, CADIC, CBPC, CMNC, CNCI, CWOB, DEI, FIML, IPUM, MCZ, MHNS, MZFC, NZAC, USNM), *Puranius obrienorum* (AMNH, CMNC, CWOB, MLP, MZFC), *Puranius pusillus* (MHNS, MLP, MZFC), *Puranius scaber* (AMPC, BMNH, CWOB, NZAC), *Puranius sylvanius* (AMNH, BMNH, CMNC, MLP, MZFC), *Puranius torosus* (MHNS, MLP, MZFC), *Puranius tothus* (MHNS), *Puranius tuberosus* (CWOB, MHNS, NZAC), *Puranius verrucosus* (CMNC, CWOB, MHNS, MZFC, NZAC) and *Puranius vulgaris* (AMNH, BMNH, CMNC, MHNS, MLP, MZFC).

#### Subtribe Palaechthina

Brinck, 1948 stat. n.

##### Palaechtini Brinck, 1948:

 43; Bouchard et al. 2011: 603 (incorrect original stem formation, not in prevailing usage).

##### Type genus.

*Palaechthus* C. O. Waterhouse, 1884 (by original designation, as *Palaechtus*, incorrect subsequent spelling).

##### Diagnosis.

Rostrum slender, as long as or longer than pronotum (except for some species of *Listronotus* where the rostrum is shorter than pronotum); scrobes long, deep, sharply bordered, reaching eyes; scape usually short (not reaching anterior margin of eye when resting in scrobe); pronotum usually subclyndrical or subcircular; elytra oblong-oval to elongate-oval.

##### Biology.

Most of the species of Palaechthina are associated to aquatic or semiaquatic plants, being found in wet or damp conditions (May 1970; O’Brien 1977, 1981; Marvaldi 1994; Morrone and O’Brien 2000). In contrast with the remaining Listroderini, larvae usually lead a more endophytic way of life inside the stems of several aquatic plants (Oberprieler, in press).

##### Included taxa.

This subtribe includes the genera *Anorthorhinus*, *Gunodes*, *Haversiella*, *Inaccodes*, *Listronotus*, *Neopachytychius*, *Palaechthus*, *Palaechtodes*, *Steriphus* and *Tristanodes*. *Anorthorhinus* and *Steriphus* are Australian; *Gunodes*, *Inaccodes*, *Palaechthus*, *Palaechtodes* and *Tristanodes* are distributed in the Tristan da Cunha-Gough islands; and the remaining three genera are found in the Americas: *Haversiella* and *Neopachytychius* in South America and *Listronotus* has a disjunct distribution in South and North America.

##### Key to the genera of Palaechthina

**Table:** 

1	Funicular segment 1 subequal to or shorter than 2	2
–	Funicular segment 1 longer than 2	6
2	Elytra with intervals convex; North and South America	*Listronotus*(Fig. 21)
–	Elytra with intervals flat; Tristan da Cunha-Gough islands	3
3	Small to very small (3.7–6.5 mm)	*Tristanodes*
–	Medium-sized to large (7.0–12.0 mm)	4
4	Vestiture of subcircular scales and setae; pronotum subcircular; elytra oblong-oval	*Gunodes*
–	Vestiture of seta-like scales and setae; pronotum subtrapezoidal or subclyndrical; elytra elongate-oval	5
5	Large (11.0–12.0 mm); rostral dorsal carinae absent; pronotum subtrapezoidal	*Palaechthus*
–	Medium-sized (7.0–7.5 mm); rostral dorsal carinae present; pronotum subclyndrical	*Palaechtodes*
6	Scape long (surpassing posterior margin of eye when resting in scrobe); elytra with anteapical tubercle	*Steriphus*
–	Scape short (not reaching anterior margin of eye when resting in scrobe); elytra lacking anteapical tubercle	7
7	Vestiture of seta-like scales and setae; Australia and Tristan da Cunha-Gough islands	8
–	Vestiture of subcircular scales ans setae; South America	9
8	Funicular segments 3-6 elongate; club fusiform; pronotum subclyndrical; elytra with intervals convex; Australia	*Anorthorhinus*
–	Funicular segments 3-6 globose; club inflated; pronotum subcircular; elytra with intervals flat; Tristan da Cunha-Gough islands	*Inaccodes*
9	Vestiture of subcircular scales and setae; rostral dorsal carinae present; mandibles long and narrow; antennal insertion distal; postocular lobes slightly developed; elytra oblong-oval; tibiae with spurs	*Neopachytychius*(Fig. 22)
–	Vestiture of subcircular scales only; rostral dorsal carinae absent; mandibles robust; antennal insertion at the middle of the rostrum; postocular lobes absent; elytra elongate-oval; tibiae lacking spurs	*Haversiella*(Fig. 20)

#### Anorthorhinus

Blackburn, 1890

http://species-id.net/wiki/Anorthorhinus

Anorthorhinus Blackburn, 1890: 327.
Anorthorrhinus Sharp, 1892: 148 (lapsus).

##### Type species.

*Anorthorhinus pictipes* Blackburn, 1890 (by indication, monotypy).

##### Diagnosis.

Small to very small (2.5–6.0 mm); vestiture of seta-like scales and setae; funicular segments 3-6 elongate; club fusiform; pronotum subclyndrical; elytra with intervals convex.

##### Relationships.

*Anorthorhinus* is the sister genus to the clade comprising *Haversiella*, *Neopachytychius* and the five genera from the Tristan da Cunha-Gough islands.

##### Species included.

*Anorthorhinus apicalis* Lea, 1899; *Anorthorhinus brevicornis* Lea, 1899; *Anorthorhinus pictipes* Blackburn, 1890.

##### Geographical distribution.

Australia (Oberprieler 2010).

##### Material examined.

*Anorthorhinus apicalis* (MZFC) and *Anorthorhinus pictipes* (MZFC).

#### Gunodes

Brinck, 1948

http://species-id.net/wiki/Gunodes

Gunodes Brinck, 1948: 55.

##### Type species.

*Gunodes major* Brinck, 1948.

##### Diagnosis.

Medium-sized (7.5 mm); vestiture of subcircular scales and setae; pronotum subcircular; elytra oblong-oval.

##### Relationships.

*Gunodes* is the sister genus to *Palaechthus-Paleachtodes-Tristanodes*. Oberprieler (1992) considered that the distinction between *Gunodes* and *Tristanodes* is not without doubt.

##### Species included. 

*Gunodes major* Brinck, 1948.

##### Geographical distribution.

Tristan da Cunha-Gough islands (Brinck 1948).

#### Haversiella

Schweiger, 1959

http://species-id.net/wiki/Haversiella

Fig. 20

Haversia Champion, 1918a: 185 (*non* Röwer, 1913).
Haversiella Schweiger, 1959: 42 (replacement name for *Haversia*).

##### Type species.

*Haversiella albolimbata* Champion, 1918 (by original designation).

##### Relationships.

*Haversiella* is the sister genus to *Neopachytychius*, andboth constitute the sister group to the five genera from the Tristan da Cunha-Gough islands.

##### Diagnosis.

Very small (3.0–3.9 mm); vestiture of subcircular scales only; maxillary mala lacking teeth; antennal insertion at the middle of the rostrum; pronotum subcircular; elytra elongate-oval; tibiae lacking spurs; plate of female sternum 8 reduced.

##### Species included.

*Haversiella albolimbata* (Champion, 1918).

##### Host plants.

Bryophytes (Morrone 1994d).

##### Geographical distribution.

Southern Argentina, including the Falkland Islands (Islas Malvinas), and southern Chile (Morrone 1994d; Posadas 2008, 2012).

##### Material examined.

*Haversiella albolimbata* (BMNH, MHNS, MZFC, USNM).

#### Inaccodes

Brinck, 1948

http://species-id.net/wiki/Inaccodes

Inaccodes Brinck, 1948: 52.

##### Type species.

*Inaccodes oblongus* Brinck, 1948.

##### Diagnosis.

Small (4.5 mm); vestiture of seta-like scales and setae; funicular segments 3-6 globose; club inflated; pronotum subcircular; elytra with intervals flat.

##### Relationships.

*Inaccodes* is the sister genus to the clade comprising the four remaining genera from the Tristan da Cunha-Gough islands. Oberprieler (1992) considered that the distinction between *Inaccodes* and *Tristanodes* is not without doubt.

##### Species included.

*Inaccodes oblongus* Brinck, 1948.

##### Geographical distribution.

Tristan da Cunha-Gough islands (Brinck 1948).

#### Listronotus

Jekel, 1865

http://species-id.net/wiki/Listronotus

Fig. 21

Macrops Kirby, 1837: 199 (*non* Wagler 1830, *nec* Burmeister 1835) (type species: not designated).
Hyperodes Jekel, 1865: 566 (type species: *Listroderes humilis* Gyllenhal, 1834, by original designation).
Listronotus Jekel, 1865: 566.
Anchodemus LeConte, 1876: 181 (type species: *Anchodemus hubbardi* LeConte, 1876, subsequent designation by Kuschel 1950: 14).
Lixellus LeConte, 1876: 182 (type species: *Lixellus filiformis* LeConte, 1876, by indication, monotypy).
Mascarauxia Desbrochers des Loges, 1898: 52 (type species: *Mascarauxia cyrtica* Desbrochers des Loges 1898, by indication, monotypy).
Relistrodes Brèthes, 1910: 209 (type species: *Relistrodes breyeri* Brèthes, 1910, by indication, monotypy).
Aulametopiellus Brèthes, 1926: 415 (type species: *Aulametopiellus dauci* Brèthes, 1926, by indication, monotypy).
Mascaranxia Bosq, 1935: 330 (lapsus).
Pseudhyperodes Hustache, 1939a: 49 (type species: *Pseudhyperodes elongatus* Hustache, 1939).

##### Type species. 

*Rhynchaenus caudatus* Say, 1824 (subsequent designation by Henderson 1940).

##### Diagnosis.

Very small to medium-sized (1.0–14.0 mm); vestiture of subcircular scales and setae; antennal insertion distal; funicular segment 1 subequal to or shorter than 2; postocular lobes present, well-developed; elytra oblong-oval to elongate-oval, with intervals convex.

##### Relationships.

*Listronotus* is the sister genus to *Steriphus* (Australia). In a previous analysis based only on American taxa (Morrone 1997a), *Listronotus* was considered to be the sister genus to *Neopachytychius*.

##### Species included.

*Listronotus alternatus* (Dietz, 1889); *Listronotus americanus* LeConte, 1876; *Listronotus angustatus* (Champion, 1902); *Listronotus annulipes* (Blatchley, 1925); *Listronotus anthracinus* (Dietz, 1889); *Listronotus apicalis* (Hustache, 1926); *Listronotus appendiculatus* (Boheman, 1842); *Listronotus argentinensis* (Hustache, 1926); *Listronotus arizonicus* O’Brien, 1981; *Listronotus blandus* Henderson, 1940; *Listronotus blatchleyi* Henderson, 1940; *Listronotus bonariensis* (Kuschel, 1955); *Listronotus borrichiae* O’Brien, 1981; *Listronotus bosqi* (Hustache, 1926); *Listronotus breyeri* (Brèthes, 1910); *Listronotus burkei* O’Brien, 1981; *Listronotus californicus* (Dietz, 1889); *Listronotus callosus* LeConte, 1876; *Listronotus carinatus* (Blatchley, 1928); *Listronotus carinicollis* (Hustache, 1926); *Listronotus caudatus* (Say, 1824); *Listronotus cinnamoneus* (Hustache, 1926); *Listronotus conabilis* O’Brien, 1981; *Listronotus crypticus* O’Brien, 1981; *Listronotus cryptops* (Dietz, 1889); *Listronotus cyrticus* (Desbrochers des Loges, 1898); *Listronotus dauci* (Brèthes, 1926); *Listronotus debilis* Blatchley, 1916; *Listronotus deceptus* (Blatchley, 1916); *Listronotus delumbis* (Gyllenhal, 1834); *Listronotus dietrichi* (Stockton, 1963); *Listronotus dietzi* O’Brien, 1979; *Listronotus distinctus* Henderson, 1940; *Listronotus dorsalis* (Dietz, 1889); *Listronotus dorytomoides* (Hustache, 1926); *Listronotus durangoensis* O’Brien, 1977; *Listronotus echinatus* (Dietz, 1889); *Listronotus echinodori* O’Brien, 1977; *Listronotus elegans* Van Dyke, 1929; *Listronotus elegantulus* O’Brien, 1981; *Listronotus elongatus* (Hustache, 1939); *Listronotus fasciatus* O’Brien, 1981; *Listronotus filiformis* (LeConte, 1876); *Listronotus frontalis* LeConte, 1876; *Listronotus geminatus* (Hustache, 1926); *Listronotus griseus* (Hustache, 1926); *Listronotus grypidioides* (Dietz, 1889); *Listronotus haldemani* (Burke, 1963); *Listronotus hirtellus* (Dietz, 1889); *Listronotus hoodi* (Stockton, 1963); *Listronotus hornii* (Dietz, 1889); *Listronotus hubbardi* (LeConte, 1876); *Listronotus humilis* (Gyllenhal, 1834); *Listronotus hyperodes* (Dietz, 1889); *Listronotus incompletus* (Hatch, 1971); *Listronotus ingens* Henderson, 1940; *Listronotus insignis* Henderson, 1940; *Listronotus laevis* (Hustache, 1926); *Listronotus laramiensis* (Angell, 1893); *Listronotus latinasus* (Blatchley, 1922); *Listronotus lineolaticollis* (Blanchard, 1851); *Listronotus lodingi* (Blatchley, 1920); *Listronotus lucens* (Hustache, 1926); *Listronotus lutulentus* (Boheman, 1843); *Listronotus maculatus* (Hatch, 1971); *Listronotus maculicollis* (Kirby, 1837); *Listronotus manifestus* Henderson, 1940; *Listronotus marginalis* O’Brien, 1977; *Listronotus marginicollis* (Hustache, 1926); *Listronotus marshalli* O’Brien, 1981; *Listronotus meridionalis* O’Brien, 1977; *Listronotus minutus* (Blanchard, 1851); *Listronotus montanus* (Dietz, 1889); *Listronotus nebulosus* LeConte, 1876; *Listronotus neocallosus* O’Brien, 1981; *Listronotus nevadicus* LeConte, 1876; *Listronotus nigropunctatus* (Suffrian, 1871); *Listronotus novellus* (Blatchley, 1916); *Listronotus obscurellus* (Dietz, 1889); *Listronotus obtectus* (Dietz, 1889); *Listronotus oregonensis* (LeConte, 1876); *Listronotus ornatipennis* (Blanchard, 1851); *Listronotus pallidus* O’Brien, 1981; *Listronotus palustris* Blatchley, 1916; *Listronotus pampaensis* (Voss, 1954); *Listronotus peninsularis* (Blatchley, 1916); *Listronotus plumosiventris* O’Brien, 1977; *Listronotus porcellus* (Say, 1831); *Listronotus poseyensis* (Blatchley, 1916); *Listronotus pseudosetosus* O’Brien, 1981; *Listronotus puncticollis* (Hustache, 1926); *Listronotus punctiger* LeConte, 1876; *Listronotus pusillus* (Hustache, 1926); *Listronotus rotundicollis* LeConte, 1876; *Listronotus rubtzoffi* O’Brien, 1981; *Listronotus rufomarginatus* (Hustache, 1939); *Listronotus salicorniae* O’Brien, 1981; *Listronotus scapularis* Casey, 1895; *Listronotus setosipennis* (Hustache, 1926); *Listronotus setosus* LeConte, 1876; *Listronotus similis* Henderson, 1940; *Listronotus sondondoanus* (Voss, 1954); *Listronotus sordidus* (Gyllenhal, 1834); *Listronotus sparsus* (Say, 1831); *Listronotus squamiger* (Say, 1831); *Listronotus sulcipennis* (Boheman, 1834); *Listronotus suturalis* O’Brien, 1981; *Listronotus teretirostris* (LeConte, 1857); *Listronotus testaceipes* (Champion, 1902); *Listronotus texanus* (Stockton, 1963); *Listronotus truncatus* (Hatch, 1971); *Listronotus tuberosus* LeConte, 1876; *Listronotus turbatus* O’Brien, 1981; *Listronotus vitticollis* (Kirby, 1837); *Listronotus vulgaris* (Hustache, 1926); *Listronotus wallacei* (Stockton, 1963); *Listronotus weiseri* (Hustache, 1926).

##### Host plants.

*Listronotus appendiculatus*: *Sagittaria latifolia* Willdenow (Alismataceae); *Listronotus argentinensis*: *Triticum aestivum* L. (Poaceae); *Listronotus blandus* : *Polygonum hydropiperoides* Michx. (Polygonaceae); *Listronotus bonariensis*: *Dactylis glomerata* L., *Festuca arundinacea* Schreber, *Hordeum vulgare* L., *Lolium multiflorum* L., *Lolium perenne* L., *Poa annua* L., *Triticum aestivum* L., *Zea mays* L. (Poaceae) and *Trifolium repens* L. (Fabaceae); *Listronotus borrichiae*: *Borrichia frutescens* (L.) DC (Asteraceae) and *Salvinia* sp. (Salviniaceae); *Listronotus caudatus*: *Polygonum bicorne* Raf. (Polygonaceae); *Listronotus cinnamoneus*: *Limnobium stoloniferum* (G. F. W. Meyer) Griseb. (Hydrocharitaceae); *Listronotus cryptops*: *Sagittaria lancifolia* L. (Alismataceae); *Listronotus dauci*: *Daucus carota* L. (Apiaceae); *Listronotus dietrichi*: *Dahlia* sp. (Asteraceae), *Gossypium* sp. (Malvaceae), *Persus* sp. (Lauraceae), *Phaseolus* sp. (Fabaceae), *Cenchrus* sp., *Chloris* sp., *Cynodon* sp., *Eleusine* sp., *Zea* sp. (Poaceae), *Coffea* sp. (Rubiaceae), *Lycopersicum* sp. (Solanaceae) and *Menta* sp. (Lamiaceae); *Listronotus echinodori*: *Echinodorus cordifolius* (L.) Griseb. and *Sagittaria latifolia* Willdenow (Alismataceae); *Listronotus elongatus*: *Hydrocotyle ranunculoides* L. f. (Apiaceae); *Listronotus haldemani*: *Juncus nodatus* Coville in N. L. Britton and A. Brown (Juncaceae); *Listronotus maculicollis*: *Agrostis palustris* Huds. and *Poa annua* L. (Poaceae); *Listronotus manifestus*: *Sagittaria longiloba* Engelm. ex J. G. Sm. (Alismataceae); *Listronotus marginicollis*: *Myriophyllum aquaticum* (Velloso) Verde (Haloragaceae); *Listronotus montanus*: *Triticum aestivum* L. (Poaceae); *Listronotus neocallosus*: *Sagittaria engelmanniana* J. G. Smith, *Sagittaria graminea* Michaux and *Sagittaria stagnorum* Small (Alismataceae); *Daucus carota* L. and *Petroselinum crispum* (Miller) A. W. Hill. (Apiaceae) (*Listronotus oregonensis*); *Listronotus plumosiventris*: *Sagittaria latifolia* Willdenow (Alismataceae); *Listronotus rotundicollis*: *Crinum* sp. (Amaryllidaceae); *Listronotus rubtzoffi*: *Sagittaria cuneata* Sheldon (Alismataceae); *Listronotus salicorniae*: *Salicornia virginica* L. (Amaranthaceae); *Listronotus scapularis*: *Sagittaria longiloba* Engelm. ex J. G. Sm. and *Sagittaria* sp. (Alismataceae); *Listronotus setosipennis*: *Parthenium hysterophorus* L. (Asteraceae); *Listronotus similis*: *Paspalum distichum* L. (Poaceae) and *Polygonum bicorne* Raf. (Polygonaceae); *Listronotus teretirostris*: *Eleocharis macrostachya* Britton (Cyperaceae); *Listronotus texanus*: *Daucus carota* L. (Apiaceae); *Listronotus turbatus*: *Sagittaria* sp. (Alismataceae) (Brèthes 1926; Burke 1963; Martel et al. 1976; May 1977; O’Brien 1977, 1981; Cordo and DeLoach 1982; Cordo et al. 1982; Edelson 1985; Barker 1989; Boivin et al. 1990; Kuschel 1990; Maes and O’Brien 1990; Anderson 1992; Wild et al. 1992; May 1993; Cragnolini 1994; Blodgett et al. 1997; Lanteri et al. 2002; Torres and Casey 2002; Rothwell 2003).

##### Immature stages.

*Listronotus bonariensis*(May, 1977, 1993, 1994; Marvaldi, 1998).

##### Geographical distribution.

Widespread in the Americas, from Canada to Argentina and Chile (O’Brien 1977, 1981; O’Brien and Wibmer 1982; Wibmer and O’Brien 1986; Maes and O’Brien 1990; Anderson 1992). This distribution corresponds to the Nearctic, Neotropical and Andean regions, as well as the South American and Mexican Transition Zones.

##### Material examined.

*Listronotus americanus* (BMNH), *Listronotus apicalis* (MLP), *Listronotus appendiculatus* (AMNH, BMNH), *Listronotus argentinensis* (AMNH, MACN, MLP9, *Listronotus blandus* (AMNH, BMNH), *Listronotus bonariensis* (BMNH, MHNS), *Listronotus bosqi* (BMNH, MLP, MZFC), *Listronotus breyeri* (MACN, MZFC), *Listronotus californicus* (AMNH), *Listronotus callosus* (BMNH, AMNH), *Listronotus caudatus* (BMNH, AMNH), *Listronotus cinnamoneus* (MLP), *Listronotus cryptops* (BMNH, AMNH), *Listronotus cyrticus* (AMNH, MACN, MLP), *Listronotus dauci* (MACN), *Listronotus debilis* (AMNH), *Listronotus delumbis* (BMNH, AMNH), *Listronotus dietzi* (AMNH), *Listronotus distinctus* (BMNH), *Listronotus durangoensis* (AMNH, BMNH), *Listronotus echinatus* (AMNH), *Listronotus echinodori* (AMNH, BMNH), *Listronotus elongatus* (MLP, MZFC), *Listronotus filiformis* (BMNH, AMNH), *Listronotus frontalis* (AMNH, BMNH), *Listronotus geminatus* (MACN, MLP), *Listronotus griseus* (AMNH, MACN, MLP), *Listronotus grypidioides* (AMNH), *Listronotus haldemani* (BMNH), *Listronotus hornii* (AMNH), *Listronotus hubbardi* (BMNH), *Listronotus humilis* (AMNH), *Listronotus hyperodes* (AMNH), *Listronotus incompletus* (AMNH), *Listronotus ingens* (AMNH), *Listronotus lineolaticollis* (MLP), *Listronotus lutulentus* (BMNH), *Listronotus maculicollis* (AMNH), *Listronotus manifestus *(AMNH, BMNH), *Listronotus marginalis* (BMNH), *Listronotus marginicollis* (MACN, MLP), *Listronotus meridionalis* (BMNH), *Listronotus minutus* (AMNH), *Listronotus nebulosus* (AMNH), *Listronotus novellus* (AMNH), *Listronotus oregonensis* (AMNH, BMNH, MZFC), *Listronotus ornatipennis* (MHNS), *Listronotus palustris* (AMNH, BMNH), *Listronotus plumosiventris* (BMNH), *Listronotus porcellus* (AMNH), *Listronotus puncticollis* (MLP), *Listronotus punctiger* (AMNH, BMNH), *Listronotus pusillus* (MLP, MZFC), *Listronotus rotundicollis* (AMNH, BMNH), *Listronotus rubtzoffi* (AMNH), *Listronotus rufomarginatus* (MLP), *Listronotus scapularis* (AMNH), *Listronotus setosipennis* (MLP), *Listronotus setosus* (AMNH), *Listronotus similis* (AMNH, BMNH), *Listronotus sordidus* (AMNH, BMNH), *Listronotus sparsus* (AMNH, BMNH), *Listronotus squamiger* (AMNH, BMNH), *Listronotus teretirostris* (AMNH, BMNH), *Listronotus texanus* (AMNH), *Listronotus truncatus* (AMNH), *Listronotus tuberosus* (AMNH, BMNH), *Listronotus vitticollis* (AMNH) and *Listronotus vulgaris* (MLP).

#### Neopachytychius

Hustache, 1939

http://species-id.net/wiki/Neopachytychius

Fig. 22

Neopachytychius Hustache, 1939b: 55.
Pernotaris Voss, 1943: 232 (type species: *Pernotaris squamiger* Voss, 1943 [= *Neopachytychius squamosus* Hustache, 1939]).

##### Type species.

*Neopachytychius squamosus* Hustache, 1939.

##### Diagnosis.

Small (3.8–6.5 mm); vestiture of subcircular scales and setae; mandible and pharyngeal process long and narrow; rostral dorsal carinae present; antennal insertion distal; postocular lobes slightly developed; elytra oblong-oval.

##### Relationships.

*Neopachytychius* is the sister genus to *Haversiella*, andboth constitute the sister group to the five genera from the Tristan da Cunha-Gough islands. In a previous analysis based only on American genera (Morrone 1997a), *Neopachytychius* was considered to be the sister genus to *Listronotus*.

##### Species included.

*Neopachytychius squamosus* Hustache, 1939.

##### Geographical distribution.

Neotropical region, in Argentina, Bolivia, Chile and Uruguay (Marvaldi 1994).

##### Material examined.

*Neopachytychius squamosus* (FIML, IADIZA, MACN, MHNS, MLP, MZFC).

#### Palaechthus

C. O. Waterhouse, 1884

http://species-id.net/wiki/Palaechthus

Palaechthus C. O. Waterhouse, 1884: 277.
Palaechtus Brinck, 1948: 47 (lapsus).

##### Type species.

*Palaechthus glabratus* Waterhouse, 1884 (subsequent designation by Brinck 1948).

##### Diagnosis.

Medium-sized (11.0–12.0 mm); vestiture of seta-like scales and setae; rostral dorsal carinae absent; pronotum subtrapezoidal.

##### Relationships.

*Palaechthus* is the sister genus to both *Paleachtodes and Tristanodes*. Oberprieler (1992) considered that the distinction between *Palaechthus* and *Palaechtodes* needs to be reevaluated.

##### Species included.

*Palaechthus glabratus* C. O. Waterhouse, 1884.

##### Geographical distribution.

Tristan da Cunha-Gough islands (Brinck 1948).

##### Material examined.

*Palaechthus glabratus* (BMNH).

#### Palaechtodes

Brinck, 1948

http://species-id.net/wiki/Palaechtodes

Palaechtodes Brinck, 1948: 50.

##### Type species.

*Palaechthus cossonoides* C. O. Waterhouse, 1884 (by original designation).

##### Diagnosis.

Medium-sized (7.0–7.5 mm); vestiture of seta-like scales and setae; rostral dorsal carinae present; pronotum subclyndrical.

##### Relationships.

*Palaechtodes* is the sister genus to both *Paleachthus and Tristanodes*. Oberprieler (1992) considered that the distinction between *Palaechtodes* and *Palaechthus* needs to be reevaluated.

##### Species included.

*Palaechtodes cossonoides* (C. O. Waterhouse, 1884).

##### Geographical distribution.

Tristan da Cunha-Gough islands (Brinck 1948).

##### Material examined.

*Palaechtodes cossonoides* (BMNH).

#### Steriphus

Erichson, 1842

http://species-id.net/wiki/Steriphus

Steriphus Erichson, 1842: 190.
Desiantha Pascoe, 1870: 193 (type species: *Desiantha caudata* Pascoe, 1870, subsequent designation by Zimmerman 1994: 697).
Brexius Pascoe, 1870: 201 (type species: *Brexius murinus* Pascoe, 1870, subsequent designation by Zimmerman 1994: 697).
Dryopais Broun, 1885: 387 (type species: *Dryopais variabilis* Broun, 1885, by indication, monotypy).
Xerostygnus Broun, 1903: 79 (type species: *Xerostygnus binodulus* Broun, 1903, by indication, monotypy).

##### Type species.

*Steriphus solidus* Erichson, 1842 (by indication, monotypy).

##### Diagnosis.

Small to very small (3.0–6.5 mm); vestiture of subcircular scales and setae; scape long (surpassing posterior margin of eye when resting in scrobe); elytra with anteapical tubercle.

##### Relationships.

*Steriphus* is the sister genus to the American genus *Listronotus*.

##### Species included.

*Steriphus albidoparsus* (Lea, 1928); *Steriphus alpinus* (Lea, 1928); *Steriphus angusticollis* (Pascoe, 1870); *Steriphus ascitus* (Pascoe, 1876); *Steriphus binodulus* Broun, 1903; *Steriphus caudatus* (Pascoe, 1870); *Steriphus curvisetosus* (Lea, 1928); *Steriphus diversipes* (Pascoe,1870); *Steriphus humeralis* (Lea, 1928); *Steriphus incotaminatus* (Lea, 1899); *Steriphus inermis* (Lea, 1928); *Steriphus irrasus* (Lea, 1899); *Steriphus longus* (Lea, 1928); *Steriphus major* (Blackburn, 1890); *Steriphus mecaspis* (Lea, 1899); *Steriphus metallicus* (Lea, 1928); *Steriphus mucronatus* (Lea, 1928); *Steriphus murinus* (Pascoe, 1870); *Steriphus parvicornis* (Lea, 1928); *Steriphus parvonigrus* (Lea, 1928); *Steriphus parvus* (Blackburn, 1890); *Steriphus pullus* (Broun, 1910); *Steriphus sericeus* (Blackburn, 1890); *Steriphus solidus* Erichson, 1842; *Steriphus stenoderes* (Lea, 1928); *Steriphus variabilis* (Broun, 1885); *Steriphus vittatus* (Blackburn, 1893).

##### Host plants.

*Steriphus ascitus*: *Baumea articulata* (R. Br.) Blake, *Baumea rubiginosa* (Spreng.) Boeck., *Scirpus fluviatilis* (Torr.) Sojak (Cyperaceae) and *Typha orientalis* C. B. Presl. (Typhaceae); *Steriphus diversipes*: *Medicago sativa* L. (Fabaceae) and *Rumex acetosella* L. (Polygonaceae); *Steriphus variabilis*: *Cotula* spp. (Asteraceae), *Dichondra* sp. (Convolvulaceae) and *Myriophyllum* sp. (Haloragaceae) (May, 1977; Kuschel, 1990).

##### Immature stages.

*Steriphus ascitus*, *Steriphus caudatus*, *Steriphus diversipes* and *Steriphus variabilis* (May, 1970, 1977, 1993, 1994).

##### Geographical distribution.

Australia and New Zealand (Schenkling and Marshall 1931; Kuschel 1972, 1990; May 1977; Zimmerman 1994).

##### Material examined.

*Steriphus ascitus* (MZFC) and *Steriphus variabilis* (MZFC).

#### Tristanodes

Brinck, 1948

http://species-id.net/wiki/Tristanodes

Tristanodes Brinck, 1948: 58.

##### Type species.

*Tristanodes craterophilus* Brinck, 1948.

##### Diagnosis.

Small to very small (3.7–6.5 mm); vestiture of seta-like scales and setae; pronotum subcylindrical.

##### Relationships.

*Tristanodes* is the sister genus to both *Palaechthus* and *Palaechtodes*. Oberprieler (1992) considered that the distinction between *Tristanodes, Gunodes and Inaccodes* is not without doubt.

##### Species included.

*Tristanodes attai* Brinck, 1948; *Tristanodes conicus* Brinck, 1948; *Tristanodes craterophilus* Brinck, 1948; *Tristanodes echinatus* Brinck, 1948; *Tristanodes insolidus* Brinck, 1948; *Tristanodes integer* Brinck, 1948; *Tristanodes medius* Brinck, 1948; *Tristanodes minor* Brinck, 1948; *Tristanodes reppetonis* Brinck, 1948; *Tristanodes scirpophilus* Brinck, 1948; *Tristanodes sivertseni* Brinck, 1948.

##### Immature stages.

*Tristanodes scirpophilus* (Kuschel, 1962).

##### Geographical distribution.

Tristan da Cunha-Gough islands (Brinck 1948; Kuschel 1962).

##### Material examined.

*Tristanodes attai* (BMNH) and *Tristanodes* spp. (BMNH).

#### Falklandiina subtr. n.

##### Type genus.

*Falklandius* Enderlein, 1907.

##### Diagnosis.

Small to very small (except *Liparogetus* and some species of *Gromilus*, which are medium-sized); rostrum stout, shorter than pronotum (except *Gromilus* and *Nestrius*, with relatively stout, medium-sized rostrum); pterygiae auriculate, exposed (Fig. 5); scrobes short, ill-defined, broad; eyes usually flat; postocular lobes usually absent (except *Gromilus* and *Falklandiopsis*); pronotum usually subcircular or subcylindrical; metanepisternal suture usually posteriorly fused or obliterated; elytra oblong-oval.

##### Included taxa.

This new subtribe, which basically corresponds to the *Falklandius* generic group of Morrone (1997a), includes the genera *Falklandiellus*, *Falklandiopsis*, *Falklandius*, *Gromilus*, *Lanteriella*, *Liparogetus*, *Nestrius* and *Telurus*. The genera *Gromilus*, *Liparogetus* and *Nestrius* are distributed in New Zealand, whereas the five remaining genera are South American, distributed in the Subantarctic subregion of the Andean region (*sensu* Morrone, 2006).

##### Key to the genera of Falklandiina

**Table:** 

1	Scrobes lateral	2
–	Scrobes dorsolateral to dorsal (Fig. 5)	6
2	Eyes transverse (Fig. 8); female elytral apex not produced; female ventrites 3 and 4 combined shorter than 5	3
–	Eyes subcircular (Fig. 5); female elytral apex produced; female ventrites 3 and 4 combined longer than 5	*Telurus*(Fig. 24)
3	Eyes slightly convex; postocular lobes absent	4
–	Eyes flat; postocular lobes slightly developed	5
4	Vestiture of setae only; rostrum very short, stout; rostral dorsal carinae absent; scrobes short, ill-defined; eyes dorsal; scape medium-sized (reaching eye when resting in scrobe); funicular segments 3-6 globose; pronotum subcircular; elytra with humeral tubercles; femora subcylindrical, markedly clavate; southern South America	*Falklandiopsis*(Fig. 24)
–	Vestiture of seta-like scales and setae; rostrum medium-sized, relatively stout; rostral dorsal carinae present; scrobes long, deep, sharply bordered, reaching eyes; eyes lateral; scape long (surpassing posterior margin of eye when resting in scrobe); funicular segments 3-6 elongate; pronotum subcylindrical; elytra lacking humeral tubercles; femora subcylindrical, clavate; New Zealand	*Gromilus*(Fig. 26)
5	Very small (2.6–3.5 mm); vestiture of subcircular scales and setae; rostrum lacking dorsal carinae; antennal insertion distal; club fusiform; pronotum transverse; metanepisternal suture posteriorly fused or obliterated; elytra with series of declivital tubercles; tibiae with spurs; southern South America	*Falklandiellus*(Fig. 23)
–	Medium-sized (6.0–10.0 mm); vestiture of seta-like scales and setae; rostrum with dorsal carinae; antennal insertion at the middle of the rostrum; club inflated; pronotum subquadrate; metanepisternal suture complete; elytra lacking series of declivital tubercles; tibiae lacking spurs; New Zealand	*Liparogetus*
6	Rostrum relatively stout, medium-sized, with dorsal carinae; eyes lateral; funicular segment 2 elongate; pronotum subcylindrical; scutellum not visible; New Zealand	*Nestrius*
–	Rostrum very short, stout, lacking dorsal carinae; eyes dorsal; funicular segment 2 globose; pronotum subcircular; scutellum visible; southern South America	7
7	Vestiture of seta-like scales and setae; eyes small; club inflated; pronotum with disc rugose; elytra with intervals convex; femora subcylindrical; tibiae subcylindrical	*Falklandius*(Fig. 25)
–	Vestiture of setae only; eyes very small, microphthalmic; club fusiform; pronotum with disc smooth, polished; elytra with intervals flat; femora dorsoventrally compressed; tibiae apically expanded	*Lanteriella*(Fig. 27)

#### Falklandiellus

Kuschel, 1950

http://species-id.net/wiki/Falklandiellus

Fig. 23

Falklandiellus Kuschel, 1950: 14.

##### Type species.

*Falklandius suffodens* Enderlein, 1907 (by original designation).

##### Diagnosis.

Very small (2.6–3.5 mm); vestiture of subcircular scales and setae; rostrum lacking dorsal carinae; antennal insertion distal; club fusiform; pronotum transverse; metanepisternal suture posteriorly fused or obliterated; elytra with series of declivital tubercles; tibiae with spurs.

##### Relationships.

*Falklandiellus* is the sister genus to *Telurus-Nestrius-Falklandius-Lanteriella*.

##### Species included.

*Falklandiellus suffodens* (Enderlein, 1907).

##### Host plants.

Bryophytes (Morrone 1995a).

##### Geographical distribution.

Andean region (Subantarctic subregion), in southern Argentina, including the Falkland Islands (Islas Malvinas), and southern Chile (Morrone 1995a; Posadas 2008, 2012).

##### Material examined.

*Falklandiellus suffodens* (BMNH, CADIC, MACN, MLP, MZFC, USNM, ZMHU).

#### Falklandiopsis

Morrone and Anderson, 1995

http://species-id.net/wiki/Falklandiopsis

Fig. 24

Falklandiopsis Morrone and Anderson, 1995: 5.

##### Type species.

*Falklandius magellanicus* Morrone, 1992.

##### Diagnosis.

Very small (3.5–4.0 mm); vestiture of setae only; rostrum very short, stout; rostral dorsal carinae absent; scrobes short, ill-defined; eyes dorsal; scape medium-sized (reaching eye when resting in scrobe); funicular segments 3-6 globose; pronotum subcircular; elytra with humeral tubercles; femora subcylindrical, markedly clavate.

##### Relationships.

*Falklandiopsis* is the sister genus to both *Liparogetus* and the clade *Falklandiellus-Telurus-Nestrius-Falklandius-Lanteriella*.

##### Species included.

*Falklandiopsis magellanica* (Morrone, 1992).

##### Host plants.

*Nothofagus betuloides* (Mirb.) Oerst. (Nothofagacae) (Morrone 1992b).

##### Geographical distribution.

Andean region (Subantarctic subregion), in southern Chile (Morrone 1992b; Posadas 2012).

##### Material examined.

*Falklandiopsis magellanica* (MLP, MZFC, NZAC, ZMHU).

#### Falklandius

Enderlein, 1907

http://species-id.net/wiki/Falklandius

Fig. 25

Falklandius Enderlein, 1907: 65.

##### Type species.

*Falklandius brachyomma* Enderlein, 1907 (= *Otiorhynchus antarcticus* Stierlin, 1903) (by original designation).

##### Diagnosis.

Small to very small (1.9–6.1 mm); vestiture of seta-like scales and setae; eyes small; club inflated; pronotal disc rugose; elytra with intervals convex.

##### Relationships.

*Falklandius* is the sister genus to *Lanteriella*, asfound in a previous analysis (Morrone, 1997a).

##### Species included.

*Falklandius antarcticus* (Stierlin, 1903); *Falklandius chilensis* Morrone and Anderson, 1995; *Falklandius goliath* Morrone, 1992; *Falklandius kuscheli* Morrone, 1992; *Falklandius peckorum* Morrone and Anderson, 1995; *Falklandius turbificatus* Enderlein, 1907.

##### Host plants.

*Falklandius antarcticus*: *Callitriche* sp. (Callitrichaceae), *Myrteola nummularia* (Poir.) O. Berg (Myrtaceae), *Nothofagus antarctica* (G. Forster) Oerst. (Nothofagacae) and *Poa flabellata* (Lam.) Raspail (Poaceae) (Morrone, 1992b); *Falklandius turbificatus*: *Myrteola nummularia* (Poir.) O. Berg (Myrtaceae) (Morrone, 1992b).

##### Geographical distribution.

Andean region (Subantarctic subregion), in southern Argentina, including the Falkland Islands (Islas Malvinas) and southern Chile (Morrone 1992b; Morrone and Anderson 1995; Posadas 2008, 2012).

##### Material examined.

*Falklandius antarcticus* (AMPC, BMNH, CADIC, CMNC, CWOB, MACN, MHNS, MZFC, USNM), *Falklandius chilensis* (AMNH, BMNH, CMNC, CWOB, FMNH, MLP, MZFC, USNM), *Falklandius goliath* (BMNH), *Falklandius kuscheli* (BMNH), *Falklandius peckorum* (AMNH, BMNH, CMNC, CWOB, FMNH, MLP, MZFC, USNM), *Falklandius turbificatus* (BMNH) and *Falklandius* spp. (MZFC).

#### Gromilus

Blanchard, 1853

http://species-id.net/wiki/Gromilus

Fig. 26

Gromilus Blanchard, 1853: 208.
Clypeorhynchus Sharp, 1883: 26 (type species: *Clypeorhynchus gracilipes* Sharp, 1883, by indication, monotypy).
Clypeorrhynchus Kirby, 1885: 100 (unjustified emendation).
Dacnophylla Broun, 1893a: 1471 (type species: *Dacnophylla setosa* Broun, 1893a, by indication, monotypy).
Hycanus Broun, 1905: 545 (type species: *Hycanus cockaynei* Broun, 1905, by indication, monotypy).
Stilbodiscus Broun, 1909: 117 (type species: *Stilbodiscus setarius* Broun, 1909, by indication, monotypy).
Phygothalpus Broun, 1913: 117 (type species: *Phygothalpus sulcicollis* Broun, 1913, by indication, monotypy).
Heteromias Broun, 1913: 120 (*non* Faust, 1897) (type species: *Heteromias foveirostris* Broun, 1913, by indication, monotypy).
Pseudohycanus Brookes, 1951: 57 (type species: *Pseudohycanus fallai* Brookes, 1951).

##### Type species.

*Gromilus insularis* Blanchard, 1853 (by indication, monotypy).

##### Diagnosis.

Small to medium-sized (3.5–7.5 mm); vestiture of seta-like scales and setae; rostrum medium-sized, relatively stout; rostral dorsal carinae present; scrobes long, deep, sharply bordered, reaching eyes; eyes lateral; scape long (surpassing posterior margin of eye when resting in scrobe); funicular segments 3-6 elongate; pronotum subcylindrical; elytra lacking humeral tubercles.

##### Relationships.

*Gromilus* is the sister genus to the remaining genera of Falklandiina. Kuschel (1964) already noted the close relationship of *Gromilus* with *Nestrius*, *Liparogetus* and *Falklandius*.

##### Species included.

*Gromilus anthracinus* (Broun, 1921); *Gromilus aucklandicus* Kuschel, 1971; *Gromilus bicarinatus* (Broun, 1921); *Gromilus bifoveatus* (Broun, 1923); *Gromilus brevicornis* (Broun, 1893); *Gromilus brounii* Morrone, 2011; *Gromilus calvulus* (Broun, 1913); *Gromilus caudatus* (Broun, 1913); *Gromilus clarulus* (Broun, 1917); *Gromilus cockaynei* (Broun, 1905); *Gromilus cordipennis* (Broun, 1893); *Gromilus cristatus* (Broun, 1893); *Gromilus dorsalis* (Broun, 1921); *Gromilus exiguus* (Brookes, 1951); *Gromilus fallai* (Brookes, 1951); *Gromilus foveirostris* (Broun, 1913); *Gromilus furvus* (Broun, 1921); *Gromilus gracilipes* (Sharp, 1883); *Gromilus granissimus* (Broun, 1917); *Gromilus halli* (Broun, 1917); *Gromilus impressus* (Broun, 1893); *Gromilus inophloeoides* (Broun, 1904); *Gromilus insularis* Blanchard, 1853; *Gromilus kuschelii* Morrone, 2011; *Gromilus laqueorum* Kuschel, 1964; *Gromilus majusculus* (Broun, 1915); *Gromilus merus* (Broun, 1917); *Gromilus narinosus* Kuschel, 1971; *Gromilus nitidellus* (Broun, 1917); *Gromilus nitidulus* (Broun, 1915); *Gromilus nodiceps* (Broun, 1914); *Gromilus philpotti* (Broun, 1917); *Gromilus setosus* (Broun, 1893); *Gromilus sparsus* (Broun, 1921); *Gromilus striatus* (Broun, 1915); *Gromilus sulcicollis* (Broun, 1913); *Gromilus sulcipennis* (Broun, 1917); *Gromilus tenuiculus* (Broun, 1921); *Gromilus thoracicus* (Broun, 1893); *Gromilus variegatus* (Broun, 1893); *Gromilus veneris* (Kirsch, 1877).

##### Host plants.

*Gromilus fallai*: *Blechnum capense* Burm. f. (Blechnaceae); *Gromilus insularis*: *Colobanthus* sp. (Caryophyllaceae), *Pleurophyllum* sp. (Asteraceae), *Poa litorosa* Cheeseman (Poaceae), *Polystichum vestitum* (G. Forst.) C. Presl. (Dryopteridaceae), *Pleurophyllum criniferum* Hook. f. (Asteraceae), *Stilbocarpa polaris* (Homb. and Jacq.) Gray (Araliaceae) and *Tillaea moschata* DC (Crassulaceae); *Gromilus setosus*: *Blechnum* sp. (Blechnaceae) and *Gahnia* sp. (Cyperaceae); *Gromilus veneris*: *Blechnum capense* (L.) Schlecht. (Blechnaceae), *Polystichum* sp. (Dryopteridaceae) and *Pteris* sp. (Pteridaceae); *Gromilus thoracicus*: *Anisotome latifolia* Hook. f. (Apiaceae), *Bulbinella* sp. (Liliaceae), *Cotula plumosa* Hook. f. and *Pleurophyllum criniferum* Hook. f. (Asteraceae) and *Poa litorosa* Cheeseman (Poaceae) (May 1977, 1993; Kuschel 1964, 1971, 1990).

**Immature stages.** *Gromilus exiguus*, *Gromilus insularis*, *Gromilus thoracicus* and *Gromilus veneris* (May, 1971).

##### Geographical distribution.

New Zealand (Schenkling and Marshall 1929; Kuschel 1964, 1971, 1990).

##### Material examined. 

*Gromilus gracilipes* (MZFC), *Gromilus insularis* (MZFC), *Gromilus laqueorum* (MZFC). *Gromilus merus* (MZFC), *Gromilus nitidellus* (MZFC) and *Gromilus veneris* (MZFC).

#### Lanteriella

Morrone, 1992

http://species-id.net/wiki/Lanteriella

Fig. 27

Lanteriella Morrone, 1992b: 167.

##### Type species.

*Lanteriella microphtalma* Morrone, 1992.

##### Diagnosis.

Very small (3.4–3.8 mm); vestiture of setae only; eyes very small, microphthalmic; pronotal disc smooth, polished; femora dorsoventrally compressed; tibiae apically expanded.

##### Relationships.

*Lanteriella* is the sister genus to *Falklandius*, asfound in a previous analysis (Morrone, 1997a).

##### Biology.

The only species of this genus was hypothesized to live in litter or soil (Morrone 1992b).

##### Species included.

*Lanteriella microphtalma* Morrone, 1992.

##### Geographical distribution.

Andean region (Subantarctic subregion), in the Falkland Islands (Islas Malvinas) (Morrone 1992b; Posadas 2008).

##### Material examined.

*Lanteriella microphtalma* (BMNH).

**Figures 27–35.**
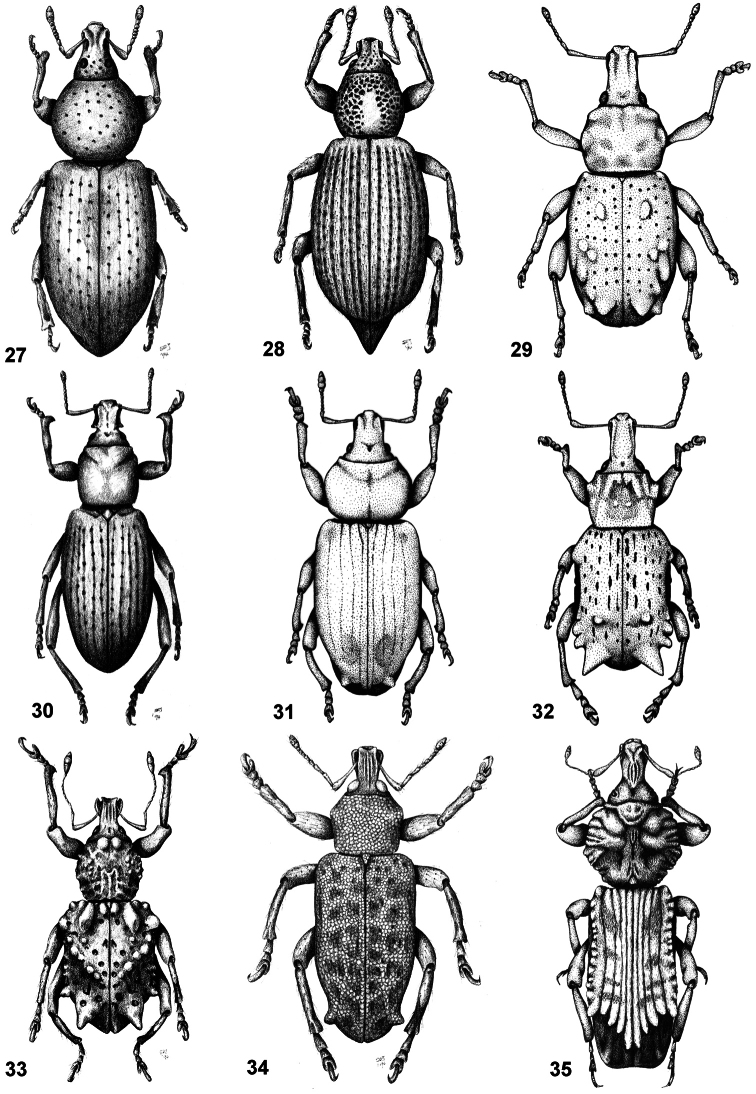
Habitus of representative Listroderini. **27** *Lanteriella microphtalma* **28** *Telurus caudiculatus* **29** *Acrorius papallacta* **30** *Acrostomus bruchi* **31** *Antarctobius lacunosus* **32** *Germainiellus dentipennis* **33** *Lamiarhinus aelficus* **34** *Listroderes annulipes* **35** *Philippius superbus*.

**Figure 36.**
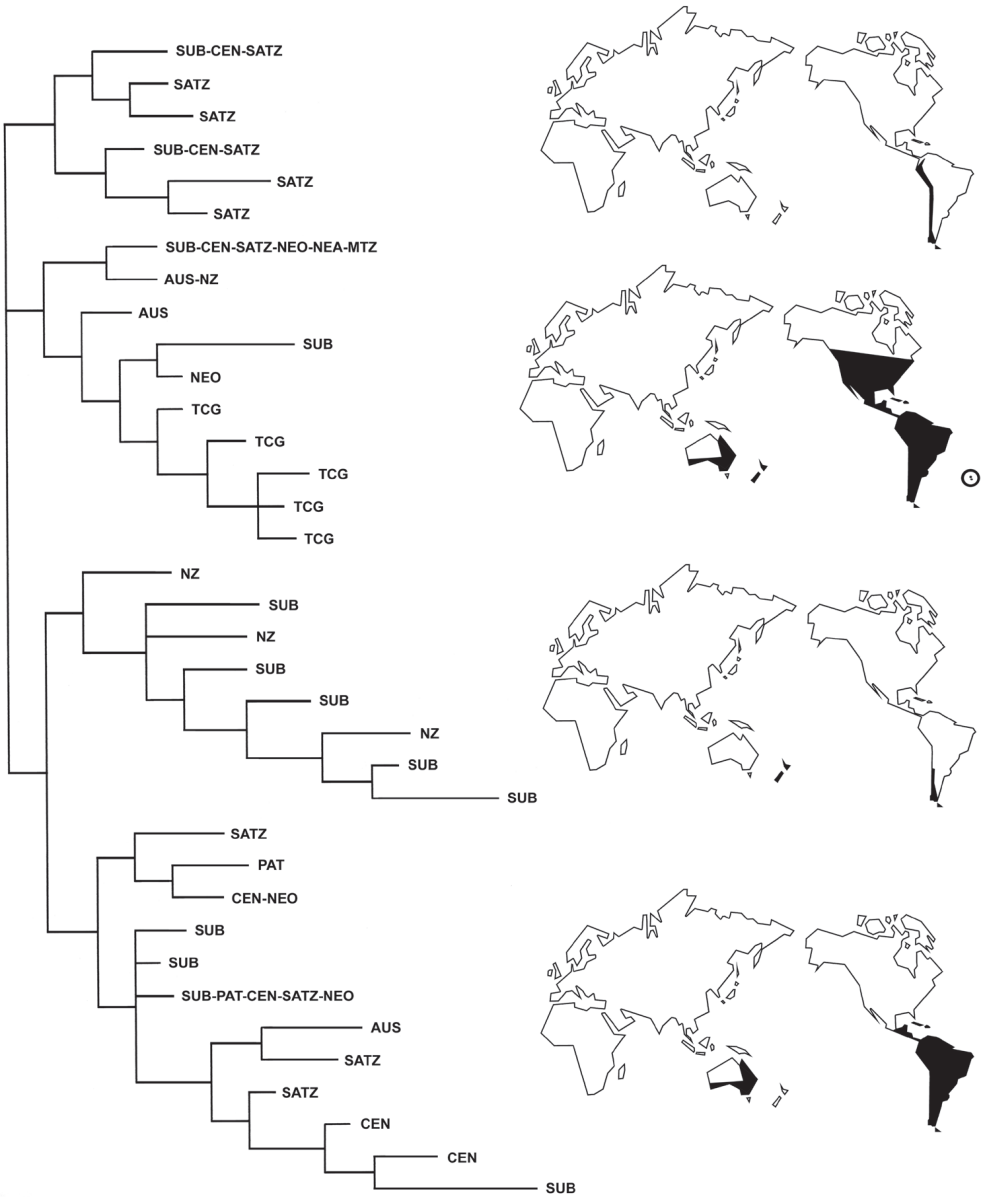
Taxon-area cladogram of the tribe Listroderini, with the geographical distribution of the subtribes represented on maps. AUS, Australia; CEN, Central Chilean subregion; MTZ, Mexican Transition Zone; NEA, Nearctic region; NEO, Neotropical region; NZ, New Zealand; PAT, Patagonian subregion; SATZ, South American Transition Zone; SUB, Subantarctic subregion; TCF, Tristan da Cunha-Gough islands.

#### Liparogetus

Broun, 1915

http://species-id.net/wiki/Liparogetus

Liparogetus Broun, 1915: 331.

##### Type species.

*Liparogetus sulcatissimus* Broun, 1915 (by indication, monotypy).

**Diagnosis.** Small to medium-sized (6.0–10.0 mm); vestiture of seta-like scales and setae; rostrum with dorsal carinae; antennal insertion at the middle of the rostrum; club inflated; pronotum subquadrate; metanepisternal suture complete; tibiae lacking spurs.

##### Relationships.

*Liparogetus* is the sister genus to both *Falklandiopsis* and the clade *Falklandiellus-Telurus-Nestrius-Falklandius-Lanteriella*.

##### Species included.

*Liparogetus sulcatissimus* Broun, 1915.

##### Geographical distribution.

New Zealand (Alonso-Zarazaga and Lyal 1999).

##### Material examined.

*Liparogetus sulcatissimus* (MZFC).

#### Nestrius

Broun, 1893

http://species-id.net/wiki/Nestrius

Nestrius Broun, 1893a: 1480.
Phyllodytes Broun, 1893a: 1479 (*non* Wagler 1830, *nec* Gistel 1848, *nec* Finsch 1873) (type species: *Phyllodytes foveatus* Broun, 1893a, by indication, monotypy).
Plotnus Broun, 1893a: 1481 (type species: *Phyllodytes ovithorax* Broun, 1893a, by indication, monotypy).
Proboscoelus Broun, 1909: 55 (type species: *Proboscoelus sculpturatus* Broun, 1909, by indication, monotypy).
Drymaria Broun, 1909: 56 (type species: *Drymaria cilipes* Broun, 1909, by indication, monotypy).
Phyllodytesius Schenkling & Marshall, 1929: 57 (replacement name for *Phyllodytes* Broun).

##### Type species.

*Nestrius serripes* Broun, 1893a (by indication, monotypy).

##### Diagnosis.

Small to very small (2.8–5.0 mm); vestiture of seta-like scales and setae; rostrum relatively stout, medium-sized, with dorsal carinae; eyes lateral; funicular segment 2 elongate; pronotum subcylindrical; scutellum not visible.

##### Relationships.

*Nestrius* is the sister genus to *Falklandius-Lanteriella*, confirming Kuschel’s (1964) suggestion that it was intermediate between *Gromilus* and *Falklandius*.

##### Species included.

*Nestrius bifurcus* Kuschel, 1964; *Nestrius cilipes* Broun, 1909; *Nestrius crassicornis* Broun, 1915; *Nestrius foveatus* (Broun, 1893); *Nestrius hudsoni* Marshall, 1953; *Nestrius irregularis* (Broun, 1910); *Nestrius laqueorum* Kuschel, 1964; *Nestrius ovithorax* (Broun, 1893); *Nestrius prolixus* Broun, 1917; *Nestrius serripes* Broun, 1893; *Nestrius sculpturatus* (Broun, 1909); *Nestrius simmondsi* Broun, 1921; *Nestrius sulcirostris* Broun, 1917; *Nestrius zenoscelis* Broun, 1921.

##### Geographical distribution.

New Zealand (Schenkling and Marshall 1929; Kuschel 1964, 1971).

##### Material examined.

*Nestrius foveatus* (MZFC) and *Nestrius sculpturatus* (MZFC).

#### Telurus

Kuschel, 1955 rev. placement

http://species-id.net/wiki/Telurus

Fig. 28

Telurus Kuschel, 1955: 288.

##### Type species.

*Antarctobius laticauda* Champion, 1918 (= *Telurus dissimilis* [Fairmaire, 1885]) (by original designation).

##### Diagnosis.

Small (3.9–6.5 mm); vestiture of setae only; eyes subcircular, slightly convex; female elytral apex produced; female ventrites 3 and 4 combined longer than 5.

##### Relationships.

*Telurus* is closely related to *Falklandius*-*Lanteriella*, as found in a previous analysis (Morrone 1997a), and to *Nestrius*, from New Zealand. Based on the presence of small scars (due to deciduous cusps) on the mandibles of *Telurus caudiculatus*, Oberprieler (2010) excluded the genus from Listroderini and transferred it to Cylydrorhininae (Entiminae). Future molecular analyses are required to corroborate its precise placement.

##### Species included.

*Telurus caudiculatus* Morrone and Anderson, 1995; *Telurus dissimilis* (Fairmaire, 1885).

##### Geographical distribution.

Andean region (Subantarctic subregion), in southern Chile (Morrone and Anderson 1995; Posadas 2012).

##### Material examined.

*Telurus caudiculatus* (AMNH, BMNH, CMNC, CNCI, CWOB, MCZ, MHNS, MLP, MZFC, USNM, ZMC) and *Telurus dissimilis* (BMNH, IPUM, MHNS, MZFC, NZAC).

#### Subtribe Listroderina

LeConte, 1876

##### Type genus.

*Listroderes* Schönherr, 1826.

##### Diagnosis.

Rostrum relatively stout, medium-sized, shorter than pronotum; scrobes short, ill-defined, broad; funicular segmen 1 longer than 2; elytra usually oblong-oval (subrectangular in *Lamiarhinus* and *Philippius*), with intervals convex and with anteapical tubercle (except for *Rupanius*).

##### Included taxa.

This subtribe, representing the listroderines in the strictest sense, includes the genera *Acroriellus*, *Acrorius*, *Acrostomus*, *Antarctobius*, *Germainiellus*, *Hyperoides*, *Lamiarhinus*, *Listroderes*, *Methypora*, *Philippius*, *Rupanius* and *Trachodema*. In a previous analysis restricted to American taxa (Morrone 1997a) most of these genera were placed as a grade basal to other listroderines. *Methypora* is distributed in Australia; and the remaining genera are South American: *Listroderes* is widely ranged in the Andean and Neotropical regions, and the other genera are found in the Andean region and the South American Transition Zone (*sensu* Morrone 2006).

##### Key to the genera of Listroderina

**Table:** 

1	Elytral disc slightly convex to flat	2
–	Elytral disc convex	6
2	Elytra oblong-oval (Fig. 10)	3
–	Elytra subrectangular (Fig. 9)	4
3	Vestiture of seta-like scales and setae; scape medium-sized (reaching eye when resting in scrobe)	*Acrorius*(Fig. 29)
–	Vestiture of scales with finger-like processes and setae; scape long (surpassing posterior margin of eye when resting in scrobe)	*Trachodema*
4	Pronotum transverse; elytra with carina on apical declivity, disc slightly convex, lacking anteapical tubercle	*Rupanius*
–	Pronotum subcircular or subcylindrical; elytra lacking carina on apical declivity, disc flat, with anteapical tubercle	*5*
5	Large (17.5–22.8 mm); mandibles with 3-4 setae; pronotum subcircular, wider than elytra, with tubercles; scutellum not visible; elytra fused along interelytral suture, with series of declivital tubercles; female elytral apex not produced; tibiae lacking spurs; tarsomeres 3 subcylindrical (Fig. 12); southern South America	*Philippius*(Fig. 35)
–	Small (4.0–7.0 mm); mandibles with 2 setae; pronotum subcylindrical, narrower than elytra, lacking tubercles; scutellum visible; elytra not fused along interelytral suture, lacking series of declivital tubercles; female elytral apex produced; tibiae with spurs; tarsomeres 3 bilobed (Fig. 11); Australia	*Methypora*
6	Funicular segments 3-6 globose; pronotum lacking tubercles; elytra oblong-oval, not fused along interelytral suture	7
–	Funicular segments 3-6 elongate; pronotum with tubercles; elytra subrectangular, fused along interelytral suture	*Lamiarhinus*(Fig. 33)
7	Pronotum transverse or subquadrate; postocular lobes present	8
–	Pronotum subcircular; postocular lobes absent	*Antarctobius*(Fig. 31)
8	Integument reddish brown; epistome not raised; pronotum transverse	9
–	Integument black; epistome raised; pronotum subquadrate	*Acrostomus*(Fig. 30)
9	Vestiture of seta-like or lanceolate scales and setae; scrobal ventral tooth absent	10
–	Vestiture of subcircular scales and setae; scrobal ventral tooth usually present	*Listroderes*(Fig. 34)
10	Vestiture of seta-like scales and setae	11
–	Vestiture of lanceolate scales and setae	*Hyperoides*
11	Elytral interval 3 lacking series of three declivital tubercles	*Germaniellus*(Fig. 32)
–	Elytral interval 3 with series of three declivital tubercles	*Acroriellus*

#### Acroriellus

Morrone and Ocampo, 1995

http://species-id.net/wiki/Acroriellus

Acroriellus Morrone & Ocampo, 1995: 257.

##### Type species.

*Acroriellus viridisquamosus* Morrone and Ocampo, 1995.

##### Diagnosis.

Very small (2.5–3.8 mm); vestiture of seta-like scales and setae; elytra with small, rounded tubercles and series of three tubercles on interval 3.

##### Relationships.

*Acroriellus* is the sister genus to *Acrostomus-Hyperoides*. Originally, it was suggested that it was close to *Acrorius* (Morrone and Ocampo 1995).

##### Species included.

*Acroriellus bobi* Morrone and Ocampo, 1995; *Acroriellus carinatus* Morrone and Ocampo, 1995; *Acroriellus similaris* Morrone and Ocampo, 1995; *Acroriellus tuberculosus* Morrone and Ocampo, 1995; *Acroriellus viridisquamosus* Morrone and Ocampo, 1995; *Acroriellus vittetae* Morrone and Ocampo, 1995.

##### Geographical distribution.

South American Transition Zone (North Andean Paramo and Puna biogeographical provinces), in Colombia, Ecuador and Peru (Morrone and Ocampo 1995).

##### Material examined.

*Acroriellus bobi* (AMNH, CMNC), *Acroriellus carinatus* (CMNC), *Acroriellus similaris* (CMNC), *Acroriellus tuberculosus* (CMNC), *Acroriellus viridisquamosus* (CMNC, FMNH) and *Acroriellus vittetae* (AMNH, USNM).

#### Acrorius

Kirsch, 1889

http://species-id.net/wiki/Acrorius

Fig. 29

Acrorius Kirsch, 1889: 25.
Ocromis Sharp, 1890: 152 (lapsus).

##### Type species.

*Acrorius puncticollis* Kirsch, 1889 (by indication, monotypy).

##### Diagnosis.

Small (4.0–6.8 mm); vestiture of seta-like scales and setae; scape medium-sized (reaching eye when resting in scrobe); elytra with small, rounded tubercles.

##### Relationships.

*Acrorius* is the sister genus to *Trachodema-Lamiarhinus-Philippius*, taxa that in a previous analysis (Morrone 1997a) constituted a paraphyletic group.

##### Species included.

*Acrorius andersoni* Morrone, 1994; *Acrorius bolivianus* Ocampo and Morrone, 1996; *Acrorius cuprinus* Morrone, 1994; *Acrorius nymphalis* Morrone, 1994; *Acrorius otramas* Ocampo and Morrone, 1996; *Acrorius papallacta* Morrone, 1994; *Acrorius pillahuata* Morrone, 1994; *Acrorius plicatifrons* Morrone, 1994; *Acrorius puncticollis* Kirsch, 1889; *Acrorius sisyphus* Morrone, 1994.

##### Geographical distribution.

Bolivia, Ecuador and Peru (Morrone 1994a; Ocampo and Morrone 1996).

##### Material examined.

*Acrorius andersoni* (CMNC), *Acrorius bolivianus* (CMNC, MZFC), *Acrorius cuprinus* (CMNC), *Acrorius nymphalis* (CMNC), *Acrorius otramas* (CMNC), *Acrorius papallacta* (CMNC, MZFC), *Acrorius pillahuata* (CMNC, FMNH), *Acrorius plicatifrons* (FMNH) and *Acrorius sisyphus* (CNCI, CMNC).

#### Acrostomus

Kuschel, 1955

http://species-id.net/wiki/Acrostomus

Fig. 30

Acrostomus Kuschel, 1955: 287.

##### Type species.

*Adioristus bruchi* Hustache, 1926 (by original designation).

##### Diagnosis.

Medium-sized (7.3–13.8 mm); integument black; vestiture of seta-like scales and setae; epistome raised; scrobal ventral tooth usually present; pronotum subquadrate.

##### Relationships.

*Acrostomus* is the sister genus to *Hyperoides*.

##### Species included.

*Acrostomus bruchi* (Hustache, 1926); *Acrostomus cruralis* Kuschel, 1958; *Acrostomus foveicollis* Kuschel, 1958; *Acrostomus griseus* (Guérin-Ménéville, 1839); *Acrostomus magellanicus* Kuschel, 1958; *Acrostomus mordor* Morrone, 1994; *Acrostomus vianai* Kuschel, 1958.

##### Host plants.

*Acrostomus magellanicus* and *Acrostomus vianai*: *Azorella trifurcata* (Gaertner) Pers., *Bolax gummifera* (Lam.) Spreng. and *Mulinum spinosum* (Cav.) Pers. (Apiaceae) (Morrone, 1994b).

##### Geographical distribution.

Andean region (Patagonian subregion), in southern Argentina and southern Chile (Morrone 1994b).

##### Material examined.

*Acrostomus bruchi* (CWOB, IPCN, MACN, MLP, MZFC), *Acrostomus cruralis* (MACN, USNM), *Acrostomus foveicollis* (CBPC, CWOB, MHNS, MZFC), *Acrostomus griseus* (CWOB, FIML, IPUM, MHNS, MLP, MZFC), *Acrostomus magellanicus* (BMNH, MHNS, USNM), *Acrostomus mordor* (AMNH, MACN, MLP, MZFC) and *Acrostomus vianai* (BMNH, MHNS).

#### Antarctobius

Fairmaire, 1885

http://species-id.net/wiki/Antarctobius

Fig. 31

Antarctobius Fairmaire, 1885: 58.

##### Type species.

*Antarctobius lacunosus* Fairmaire, 1885 (subsequent designation by Morrone, 1992a).

##### Diagnosis.

Small to medium-sized (3.7–9.5 mm); vestiture of seta-like or subcircular scales and setae; pronotum subcircular; postocular lobes absent.

##### Relationships.

*Antarctobius* is closely related to *Germainiellus, Listroderes and* the clade *Methypora-Rupanius-Acrorius-Trachodema-Lamiarhinus-Philippius*. The distinction between *Antarctobius*, *Germainiellus* and *Listroderes* is not without doubt (see Morrone and Marvaldi, 1998), and future analyses may determine if they are merged into a single genus.

##### Species included.

*Antarctobius abditus* (Enderlein, 1907); *Antarctobius bidentatus* (Champion, 1918); *Antarctobius falklandicus* (Enderlein, 1907); *Antarctobius germaini* (Kolbe, 1907); *Antarctobius hyadesii* Fairmaire, 1885; *Antarctobius lacunosus* Fairmaire, 1885; *Antarctobius malvinensis* Posadas and Morrone, 2004; *Antarctobius rugirostris* Champion, 1918; *Antarctobius vulsus* (Enderlein, 1907); *Antarctobius yefacel* Morrone, 1992.

##### Host plants.

*Antarctobius abditus*: *Senecio candidans* DC (Asteraceae); *Antarctobius hyadesii*: *Senecio alloeophyllus* O. Hoffm. and *Senecio candidans* DC (Asteraceae) (Morrone, 1992a; Marvaldi, 1998).

##### Immature stages.

*Antarctobius abditus* and *Antarctobius falklandicus* (Marvaldi, 1998).

##### Geographical distribution.

Andean region (Subantarctic subregion), in southern Chile and southern Argentina, including the Falkland Islands (Islas Malvinas) (Morrone 1992a; Posadas and Morrone 2004; Posadas 2008, 2012).

##### Material examined.

*Antarctobius abditus* (BMNH), *Antarctobius bidentatus* (BMNH), *Antarctobius falklandicus* (AMPC, BMNH, MZFC), *Antarctobius germaini* (AMNH, BMNH, CADIC, CMNC, CWOB, IPUM, MHNS, MLP, MZFC), *Antarctobius hyadesii* (BPBM, CMNC, MHNS, MZFC), *Antarctobius lacunosus* (BMNH, MCZ, MHNS), *Antarctobius rugirostris* (BMNH), *Antarctobius vulsus* (BMNH, USNM) and *Antarctobius yefacel* (AMNH).

#### Germainiellus

Morrone, 1993

http://species-id.net/wiki/Germainiellus

Fig. 32

Germainiellus Morrone, 1993a: 125.

##### Type species.

*Listroderes dentipennis* Germain, 1895 (by original designation).

##### Diagnosis.

Small to medium-sized (6.0-8.4 mm); vestiture of seta-like scales and setae; pronotum transverse; postocular lobes present.

##### Relationships.

*Germainiellus* is closely related to *Antarctobius*, *Listroderes* and the clade *Methypora-Rupanius-Acrorius-Trachodema-Lamiarhinus-Philippius*. It was originally described as intermediate between *Antarctobius* and *Listroderes* (Morrone, 1993a). The distinction between *Antarctobius*, *Germainiellus* and *Listroderes* is not without doubt (see Morrone and Marvaldi, 1998), and future analyses may determine if they are merged into a single genus.

##### Species included.

*Germainiellus angulipennis* (Germain, 1895); *Germainiellus attenuatus* (Germain, 1895); *Germainiellus dentipennis* (Germain, 1895); *Germainiellus fulvicornis* (Germain, 1895); *Germainiellus laevirostris* (Germain, 1895); *Germainiellus lugens* (Germain, 1895); *Germainiellus ovatus* (Boheman, 1842); *Germainiellus philippii* (Germain, 1896); *Germainiellus planipennis* (Blanchard, 1851); *Germainiellus punctiventris* (Germain, 1895); *Germainiellus rugipennis* (Blanchard, 1851); *Germainiellus salebrosus* (Enderlein, 1907); *Germainiellus* spp. (MZFC).

##### Host plants.

*Germainiellus dentipennis* and *Germainiellus fulvicornis*: *Nothofagus* sp. (Nothofagaceae); *Germainiellus laevirostris*: *Senecio smithii* DC (Asteraceae); *Germainiellus planipennis*: *Nothofagus dombeyi* (Mirb.) Oerst. (Nothofagaceae) and *Peumus boldus* Mol. (Monimiaceae); *Germainiellus salebrosus*: *Empetrum rubrum* Vahl ex Willd. (Empetraceae) (Morrone, 1993a).

##### Geographical distribution.

Andean region (Subantarctic subregion), in southern Chile and southern Argentina, including the Falkland Islands (Islas Malvinas) (Morrone 1993a, 1994e; Posadas 2008, 2012).

##### Material examined.

*Germainiellus angulipennis* (MHNS), *Germainiellus attenuatus* (ARPC, MHNS), *Germainiellus dentipennis* (CBPC, CMNC, CWOB, MHNS, USNM), *Germainiellus fulvicornis* (AMNH, BPBM, CBCP, CMNC, CWOB, FIML, MCZ, MHNS, MLP, MZFC, USNM), *Germainiellus laevirostris* (BPBM, IPUM, MCZ, MHNS, MLP, USNM), *Germainiellus lugens* (CMNC, CWOB, IPUM, MACN, MCZ, MHNS, MZFC), *Germainiellus ovatus* (BMNH, MHNS, USNM), *Germainiellus philippii* (CMNC, DEI, MHNS, MZFC), *Germainiellus planipennis* (BMNH, CWOB, MHNS), *Germainiellus punctiventris* (MHNS), *Germainiellus rugipennis* (AMNH, BPBM, CADIC, CBCP, CMNC, MHNS, MLP, MZFC, USNM) and *Germainiellus salebrosus* (BMNH).

#### Hyperoides

Marshall, 1914

http://species-id.net/wiki/Hyperoides

Hyperoides Marshall, 1914: 236.

##### Type species.

*Hyperoides fragariae* Marshall, 1914 (by indication, monotypy).

##### Diagnosis.

Small to medium-sized (5.1–7.5 mm); vestiture of lanceolate scales and setae; postocular lobes present; elytra lacking anteapical tubercle.

##### Relationships.

*Hyperoides* is the sister genus to *Acrostomus*, contrasting with its more isolated position in a previous analysis (Morrone, 1997a).

##### Species included.

*Hyperoides balfourbrownei* (Kuschel, 1952); *Hyperoides fragariae* Marshall, 1914; *Hyperoides murinus* (Germain, 1896); *Hyperoides subcinctus* (Boheman, 1842); *Hyperoides victus* (Germain, 1896).

##### Host plants.

*Hyperoides fragariae*: *Fragaria vesca L*. (Rosaceae); *Hyperoides subcinctus*: *Senecio* sp. (Asteraceae); *Hyperoides murinus*: *Citrulus vulgaris* Schrad. (Cucurbitaceae), *Phaseolus* sp. (Fabaceae) and *Solanum tuberosum* L. (Solanaceae); *Hyperoides victus*: *Senecio bahioides* Hook. et Arn. (Asteraceae) (Morrone 1993b; Lanteri et al. 2002).

##### Geographical distribution.

Neotropical region and Andean region (Central Chilean subregion), in Argentina, Chile and Uruguay, and introduced into South Africa (Morrone 1993b).

##### Material examined.

*Hyperoides balfourbrownei* (MLP, MZFC), *Hyperoides fragariae* (BMNH, CBPC, MNHN), *Hyperoides murinus* (BMNH, CWOB, MHNS, MZFC), *Hyperoides subcinctus* (AMNH, BMNH, CBPC, CMNC, CWOB, IADIZA, MACN, MHNS, MNHN, MZFC) and *Hyperoides victus* (BMNH, CMNC, CWOB, MHNS).

#### Lamiarhinus

Morrone, 1992

http://species-id.net/wiki/Lamiarhinus

Fig. 33

Lamiarhinus Morrone, 1992c: 419.

##### Type species.

*Lamiarhinus aelficus* Morrone, 1992.

##### Diagnosis.

Small to medium-sized (5.7–6.8 mm); vestiture of seta-like scales and setae; funicular segments 3-6 elongate; pronotum with tubercles; elytra subrectangular, fused along interelytral suture.

##### Relationships.

*Lamiarhinus* is the sister genus to *Philippius*. In a previous analysis (Morrone 1997a), it was considered to be related to *Trachodema*.

##### Species included.

*Lamiarhinus aelficus* Morrone, 1992; *Lamiarhinus horridus* (Germain, 1896).

##### Host plants.

*Lamiarhinus aelficus*: *Podanthus ovatifolius* Lag. (Asteraceae) (Morrone 1992c).

##### Geographical distribution.

Andean region (Central Chilean subregion) (Morrone 1992c).

##### Material examined.

*Lamiarhinus aelficus* (CMNC, CWOB, MLP, MZFC) and *Lamiarhinus horridus* (MHNS).

#### Listroderes

Schönherr, 1826

http://species-id.net/wiki/Listroderes

Fig. 34

Listroderes Schönherr, 1823: col. 1142 (*nom. nud*.).
Listroderes Schönherr, 1826: 158.
Listroderus Erichson, 1847: 129 (lapsus).
Listoderes Kuschel, 1990: 71 (lapsus).

##### Type species.

*Listroderes costirostris* Schönherr, 1826 (by original designation, combined description).

##### Diagnosis.

Small to medium-sized (3.9–12.5 mm); vestiture of subcircular scales and setae; scrobal ventral tooth usually present.

##### Relationships.

*Listroderes* is closely related to *Antarctobius, Germainiellus and* the clade *Methypora-Rupanius-Acrorius-Trachodema-Lamiarhinus-Philippius*. The distinction between *Antarctobius*, *Germainiellus* and *Listroderes* is not without doubt (see Morrone and Marvaldi 1998), and future analyses may determine if they are merged into a single genus.

##### Species included.

*Listroderes affinis* Hustache, 1926; *Listroderes angusticeps* Blanchard, 1851; *Listroderes annulipes* Blanchard, 1851; *Listroderes apicalis* Waterhouse, 1841; *Listroderes bimaculatus* Boheman, 1842; *Listroderes brevirostris* Germain, 1895; *Listroderes brevisetis* Hustache, 1926; *Listroderes bruchi* Hustache, 1926; *Listroderes charybdis* Morrone, 1993; *Listroderes cinerarius* Blanchard, 1851; *Listroderes confusus* Hustache, 1926; *Listroderes costirostris* Schönherr, 1826; *Listroderes curvipes* Germain, 1895; *Listroderes delaiguei* Germain, 1895; *Listroderes desertorum* Germain, 1895; *Listroderes difficilis* Germain, 1895; *Listroderes elegans* Hustache, 1926; *Listroderes erinaceus* Germain, 1895; *Listroderes fallax* Germain, 1895; *Listroderes foveatus* (Lea, 1928); *Listroderes hoffmanni* Germain, 1895; *Listroderes howdenae* Morrone, 1993; *Listroderes leviculus* Kuschel, 1952; *Listroderes montanus* Germain, 1895; *Listroderes nodifer* Boheman, 1842; *Listroderes obliquus* Klug, 1829; *Listroderes obrieni* Morrone, 1993; *Listroderes paranensis* Hustache, 1926; *Listroderes punicola* Kuschel, 1949; *Listroderes pusillus* Hustache, 1926; *Listroderes robustior* Schenkling and Marshall, 1931; *Listroderes robustus* Waterhouse, 1841; *Listroderes scylla* Morrone, 1993; *Listroderes trivialis* Germain, 1895; *Listroderes tuberculifer* Blanchard, 1851; *Listroderes uruguayensis* Kuschel, 1952; *Listroderes wagneri* Hustache, 1926; *Listroderes wittei* Hustache, 1926.

##### Host plants.

*Listroderes apicalis*: *Betavulgaris* L. (Chenopodiaceae), *Helianthus annus* L. (Asteraceae) and *Triticum aestivum* L. (Poaceae); *Listroderes bimaculatus*: *Baccharis linearis* (Ruiz and Pav.) Pers. (Asteraceae) and *Puya chilensis* Molina (Bromeliaceae); *Listroderes bruchi*: *Baccharis salicifolia* (Ruiz and Pavón) Pers. and *Senecio subulatus* Don Hooker et Arnott (Asteraceae); *Listroderes cinerarius*: *Atriplex* sp. (Chenopodiaceae); *Listroderes costirostris*, *Listroderes difficilis* and *Listroderes obliquus*: *Apium graveolens* L. and *Daucus carota* L. (Apiaceae), *Brassica rapa* L., *Listroderes oleracea* L. and *Coronopus didymus* (L.) Smith (Brassicaceae), *Rumex altissimus* Wood (Polygonaceae), *Nicotiana tabacum* L. and *Solanum tuberosum* L. (Solanaceae) and *Stellaria* spp. (Caryophyllaceae); *Listroderes robustus*: *Atriplex semibaccata* R. Br. (Chenopodiaceae); *Listroderes uruguayensis*: *Hydrocotyle bonariensis* Lam. (Apiaceae) (Morrone 1993d, 1995b; Marvaldi 1998; Lanteri et al. 2002).

##### Immature stages.

*Listroderes bruchi*, *Listroderes delaiguei* and *Listroderes difficilis* (May 1977, 1993, 1994; Marvaldi 1998).

##### Geographical distribution.

Andean region (Subantarctic, Central Chilean and Patagonian subregions), South American Transition Zone and Neotropical region, in Argentina, Brazil, Chile, Paraguay, Peru and Uruguay, and introduced into Australia, Easter Island, Israel, Japan, New Zealand, South Africa, Spain and USA (Wibmer and O’Brien 1986; Kuschel 1990; Morrone 1993c-e, 1995b; Morrone 2002b; Friedman 2009; Posadas 2012).

##### Material examined.

*Listroderes affinis* (CBPC, IPCN, IPUM, MACN, MNHN), *Listroderes angusticeps* (MHNS, MNHN, MZFC), *Listroderes annulipes* (CBPC, CWOB, MHNS, MNHN, MZFC), *Listroderes apicalis* (AMNH, BMNH, CMNC, MACN, MHNS, MLP, MZFC), *Listroderes bimaculatus* (AMNH, BMNH, CMNC, CWOB, MACN, MHNS), *Listroderes brevirostris* (MHNS), *Listroderes brevisetis* (CBCP, DZUP, IPCN, MACN, MLP, MNHN), *Listroderes bruchi* (CMNC, DZUP, FIML, IADIZA, MACN, MLP, MZFC), *Listroderes charybdis* (MACN, MLP), *Listroderes cinerarius* (BMNH, CMNC, CWOB, IADIZA, MHNS, MNHN, MZFC), *Listroderes confusus* (DZUP, FIML, MACN, MLP, MNHN), *Listroderes costirostris* complex (AMNH, BMNH, CBPC, CMNC, CWOB, DZUP, FIML, GJWC, MACN, MHNS, MLP, MNHN, MZFC, MZSP, USNM), *Listroderes curvipes* (BMNH, CWOB, MHNS), *Listroderes delaiguei* (BMNH, CADIC, CWOB, IPUM, MHNS, MZFC), *Listroderes desertorum* (BMNH, CMNC, CWOB, MHNS, MZFC), *Listroderes elegans* (GJWC, MACN, MLP, MNHN), *Listroderes erinaceus* (MHNS), *Listroderes fallax* (CWOB, MHNS, MZFC), *Listroderes foveatus* (BMNH, CMNC, DZUP, FIML, GJWC, MACN, MZSP), *Listroderes hoffmanni* (BMNH, CWOB, MHNS, MZFC), *Listroderes howdenae* (CMNC, MLP, MZFC), *Listroderes leviculus* (BMNH), *Listroderes montanus* (MHNS, MZFC), *Listroderes nodifer* (BMNH, CWOB, MACN, MHNS), *Listroderes obrieni* (MHNS, MLP, MZFC), *Listroderes paranensis* (DZUP, MNHN), *Listroderes punicola* (CMNC, MHNS, MZFC), *Listroderes pusillus* (CBPC, MLP, MNHN, MZFC), *Listroderes robustior* (BMNH, CMNC, CWOB, MHNS, MLP, MZFC), *Listroderes robustus* (CMNC, CWOB, MHNS), *Listroderes scylla* (FIML, MLP), *Listroderes trivialis* (MHNS), *Listroderes tuberculifer* (CMNC, MHNS, MZFC), *Listroderes uruguayensis* (BMNH, CMNC), *Listroderes wagneri* (BMNH, MNHN) and *Listroderes wittei* (MACN, MNHN).

#### Methypora

Pascoe, 1865

http://species-id.net/wiki/Methypora

Methypora Pascoe, 1865: 416.

##### Type species.

*Methypora postica* Pascoe, 1865 (by indication, monotypy).

##### Diagnosis.

Small (4.0–7.0 mm); vestiture of subcircular scales and setae; pronotum subcylindrical, lacking tubercles; scutellum visible; elytra not fused along interelytral suture, lacking series of declivital tubercles; female elytral apex produced; tibiae with spurs.

##### Relationships.

*Methypora* is the sister genus to *Rupanius*.

##### Species included.

*Methypora postica* Pascoe, 1865 and *Methypora tibialis* Lea, 1911.

##### Geographical distribution.

Australia (Oberprieler 2010).

##### Material examined.

*Methypora postica* (BMNH).

#### Philippius

Germain, 1895

http://species-id.net/wiki/Philippius

Fig. 35

Philippius Germain, 1895: 314.

##### Type species.

*Listroderes superbus* Reed, 1872 (subsequent designation by Wibmer and O’Brien 1986).

##### Diagnosis.

Large to very large (17.5–22.8 mm); vestiture of scales with finger-like processes and setae; mandible with 3-4 setae; pronotum wider than elytra; scutellum not visible; elytra subrectangular, fused along interelytral suture; tibiae lacking spurs; tarsomeres 3 subcylindrical.

##### Relationships.

*Philippius* is the sister genus to *Lamiarhinus*.

##### Species included.

*Philippius superbus* (Reed, 1872).

##### Geographical distribution.

Andean region (Subantarctic subregion), in southern Argentina and southern Chile (Kuschel 1987; Morrone 1990).

##### Material examined.

*Philippius superbus* (IADIZA, MACN, MHNS, MLP, USNM).

#### Rupanius

Morrone, 1995

http://species-id.net/wiki/Rupanius

Rupanius Morrone, 1995c: 604.

##### Type species.

*Rupanius carinatus* Morrone, 1995c.

##### Diagnosis.

Small (5.3–6.6 mm); vestiture of seta-like scales and setae; pronotum transverse; elytra subrectangular, with carina on apical declivity, disc slightly convex, lacking anteapical tubercle.

##### Relationships.

*Rupanius* is the sister genus to *Methypora* (Australia), and both are placed in Listroderina. In a previous analysis (Morrone 1997a) *Rupanius* was placed in the *Macrostyphlus* generic group (= Macrostyphlina).

##### Species included.

*Rupanius carinatus* Morrone, 1995.

##### Geographical distribution.

South American Transition Zone (North Andean Paramo biogeographical province), in Colombia (Morrone 1995c).

##### Material examined.

*Rupanius carinatus* (CMNC).

#### Trachodema

Blanchard, 1849

http://species-id.net/wiki/Trachodema

Trachodema Blanchard, 1849: pl. 24.

##### Type species.

*Trachodema tuberculosa* Blanchard, 1849 (by indication, monotypy).

##### Diagnosis.

Small to very small (2.5–5.3 mm); vestiture of scales with finger-like processes and setae; scape long (surpassing posterior margin of eye when resting in scrobe); pronotum transverse.

##### Relationships.

*Trachodema* is the sister genus to *Lamiarhinus-Philippius*.

##### Species included.

*Trachodema paolae* Alonso-Zarazaga, 2012 and *Trachodema tuberculosa* Blanchard, 1849.

##### Host plants.

*Trachodema tuberculosa*: *Atriplex semibaccata* R. Br. (Chenopodiaceae) (Morrone 1992c).

##### Geographical distribution.

Andean region (Central Chilean subregion) (Morrone 1992c).

##### Material examined.

*Trachodema paolae* (MHNS) and *Trachodema tuberculosa* (CMNC, CWOB, DZUP, FIML, MHNS, MZFC, USNM).

### Species *inquirendae*

#### Listroderes bicallosus

(Boheman, 1859)

Cryptorhynchus bicallosus Boheman, 1859: 139.
Listroderes bicallosus ; Wibmer and O’Brien, 1986: 113.

##### Distribution.

Ecuador and Peru (Wibmer and O’Brien 1986).

#### Listroderes mus

Germain, 1895

Listroderes mus Germain, 1895: 102.

##### Distribution.

Chile (Wibmer and O’Brien 1986).

### Biogeographical Account

The geographical distribution of the genera analysed indicates that Listroderini are basically a Gondwanan taxon, with *Listronotus* being the only genus distributed in North America. All the subtribes have Andean representatives (especially in the Subantarctic subregion), each showing a different pattern:

1 Macrostyphlina: exclusively Andean, in both the Andean region (Subantarctic and Central Chilean subregions) and the South American Transition Zone.

2 Falklandiina: distributed in the Andean region (Subantarctic subregion) and New Zealand.

3 Listroderina: distributed in the Andean region (Subantarctic, Patagonian and Central Chilean subregions), the South American and Mexican Transition Zones and the Neotropical and Australian Temperate regions.

4 Palaechthina: distributed in the Andean (Subantarctic and Central Chilean subregions), Neotropical and Nearctic regions, the South American Transition Zone, the Tristan da Cunha-Gough islands, New Zealand and the Australian Temperate region.

By replacing the genera for the areas where they are distributed, a taxon-area cladogram was obtained (Fig. 36). The paralogy-free subtrees that can be obtained from this taxon-area cladogram are mostly uninformative, and the informative ones cannot be combined into a general area cladogram. Geographical paralogy is particularly evident in the Subantarctic subregion, where representatives of the four subtribes are represented, suggesting that Listroderini are an ancient Gondwanan group. Several extinction events might have obscured the relationships among the areas.

## Supplementary Material

XML Treatment for Listroderini

XML Treatment for Macrostyphlina

XML Treatment for Adioristidius

XML Treatment for Amathynetoides

XML Treatment for Andesianellus

XML Treatment for Macrostyphlus

XML Treatment for Nacodius

XML Treatment for Puranius

XML Treatment for Palaechthina

XML Treatment for Anorthorhinus

XML Treatment for Gunodes

XML Treatment for Haversiella

XML Treatment for Inaccodes

XML Treatment for Listronotus

XML Treatment for Neopachytychius

XML Treatment for Palaechthus

XML Treatment for Palaechtodes

XML Treatment for Steriphus

XML Treatment for Tristanodes

XML Treatment for Falklandiina

XML Treatment for Falklandiellus

XML Treatment for Falklandiopsis

XML Treatment for Falklandius

XML Treatment for Gromilus

XML Treatment for Lanteriella

XML Treatment for Liparogetus

XML Treatment for Nestrius

XML Treatment for Telurus

XML Treatment for Listroderina

XML Treatment for Acroriellus

XML Treatment for Acrorius

XML Treatment for Acrostomus

XML Treatment for Antarctobius

XML Treatment for Germainiellus

XML Treatment for Hyperoides

XML Treatment for Lamiarhinus

XML Treatment for Listroderes

XML Treatment for Methypora

XML Treatment for Philippius

XML Treatment for Rupanius

XML Treatment for Trachodema

XML Treatment for Listroderes bicallosus

XML Treatment for Listroderes mus
